# Microglia contribute to the propagation of Aβ into unaffected brain tissue

**DOI:** 10.1038/s41593-021-00951-0

**Published:** 2021-11-22

**Authors:** Paolo d’Errico, Stephanie Ziegler-Waldkirch, Vanessa Aires, Philippe Hoffmann, Charlotte Mezö, Daniel Erny, Laura Sebastian Monasor, Sabine Liebscher, Vidhya M. Ravi, Kevin Joseph, Oliver Schnell, Katrin Kierdorf, Ori Staszewski, Sabina Tahirovic, Marco Prinz, Melanie Meyer-Luehmann

**Affiliations:** 1 Department of Neurology, Medical Center – University of Freiburg, Freiburg, Germany; 2 Faculty of Medicine, University of Freiburg, Freiburg, Germany; 3 Faculty of Biology, University of Freiburg, Freiburg, Germany; 4 Institute of Neuropathology, Faculty of Medicine, University of Freiburg, Freiburg, Germany; 5 Berta-Ottenstein-Programme, Faculty of Medicine, University of Freiburg, Freiburg, Germany; 6 German Center for Neurodegenerative Diseases (DZNE), Munich, Germany; 7 Munich Cluster for Systems Neurology (SyNergy), Munich, Germany; 8 Institute of Clinical Neuroimmunology, Klinikum der Universität München, Ludwig-Maximilians University Munich, Martinsried, Germany; 9 Biomedical Center, Medical Faculty, Ludwig-Maximilians University Munich, Martinsried, Germany; 10 Microenvironment and Immunology Research Laboratory, Medical Center – University of Freiburg, Freiburg, Germany; 11 Department of Neurosurgery, Medical Center – University of Freiburg, Freiburg, Germany; 12 Neuroelectronic Systems, Medical Center – University of Freiburg, Freiburg, Germany; 13 Translational NeuroOncology Research Group, Medical Center – University of Freiburg, Freiburg, Germany; 14 Centre for Integrative Biological Signalling Studies, University of Freiburg, Freiburg, Germany; 15 Center for Basics in NeuroModulation (NeuroModulBasics), Faculty of Medicine, University of Freiburg, Freiburg, Germany; 16 Signalling Research Centres BIOSS and CIBSS, University of Freiburg, Freiburg, Germany

**Keywords:** Glial biology, Diseases

## Abstract

Microglia appear activated in the vicinity of amyloid beta (Aβ) plaques, but whether microglia contribute to Aβ propagation into unaffected brain regions remains unknown. Using transplantation of wild-type (WT) neurons, we show that Aβ enters WT grafts, and that this is accompanied by microglia infiltration. Manipulation of microglia function reduced Aβ deposition within grafts. Furthermore, in vivo imaging identified microglia as carriers of Aβ pathology in previously unaffected tissue. Our data thus argue for a hitherto unexplored mechanism of Aβ propagation.

## Abstract (web summary)

This study shows that Aβ from transgenic host tissue is able to enter and deposit within wild-type grafts via microglia, thus identifying microglia as carriers of Aβ deposition into previously unaffected brain tissue.

## Main

The aggregation of Aβ is an essential early trigger in Alzheimer’s disease (AD) pathogenesis that leads to neurofibrillary tangles, neuronal dysfunction and dementia^1^. Several cell types have been proposed to be causally involved in amyloid plaque formation, including microglia, owing to their close association with Aβ plaques^2–4^. As soon as Aβ plaques form in the brain, microglia establish an intimate contact with them and become reactive^5,6^. Those activated microglia have been linked to plaque growth by Aβ uptake followed by microglial cell death^7,8^. Our group and others have recently implicated microglia in Aβ seeding^9–11^, yet their role in propagating Aβ pathology remains elusive.

In support of the ‘pathogenic spread’ hypothesis^12^, previous transplantation experiments showed that Aβ derived from transgenic host tissue is able to invade and deposit in non-transgenic grafts, leading to neurodegeneration^13–15^. However, the mechanism by which Aβ spreads into the WT grafts is unknown, and a cell-mediated mechanism has not been proven so far.

**Fig. 1:**
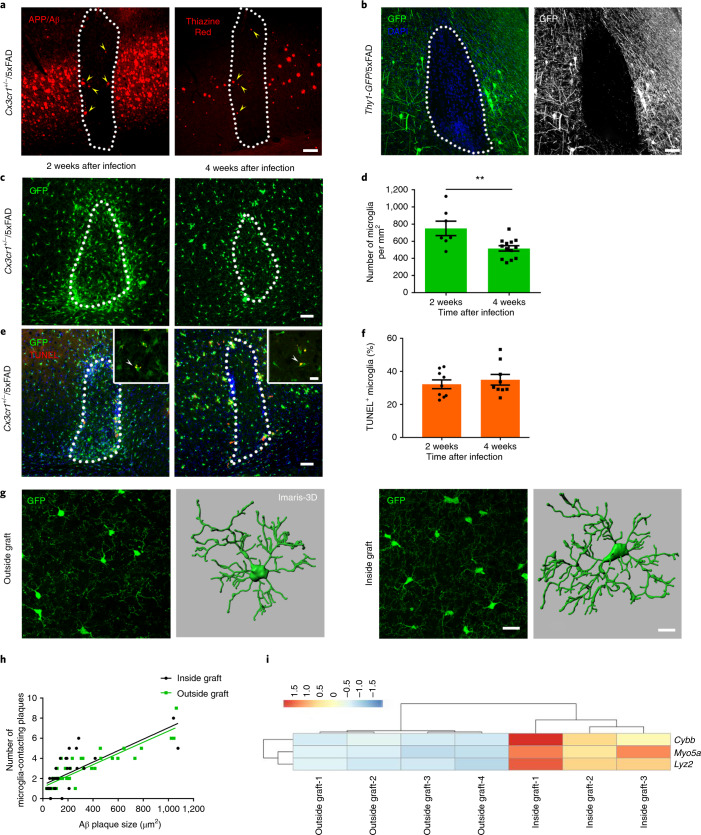
Characteristics of microglia in neuronal WT grafts. **a**, Fluorescence microscopy of a graft 4 weeks after injection into *Cx3cr1*^+/−^/5xFAD cortex. Yellow arrowheads indicate Aβ deposition (left: 6E10 in red, from *n* = 9 mice; right: Thiazine Red in red, from *n* = 4 mice) inside the graft (outlined with a white dotted line). Scale bar, 50 μm. **b**, Representative fluorescence microscopy of a graft at 4 weeks after injection in *Thy1-GFP*/5xFAD recipient mice, from *n* = 4 mice. Scale bar, 50 μm. **c**, Analysis of microglia accumulation inside grafts at 2 and 4 weeks after injection in *Cx3cr1*^+/−^/5xFAD transgenic mice. Scale bar, 50 μm. **d**, Graph shows mean (± s.e.m.) of microglia cell density inside grafts; each symbol represents one graft from *n* = 7 (2 weeks) and *n* = 13 (4 weeks) mice. Significant differences were determined using the Mann–Whitney test (**P = 0.0085). **e**, Fluorescence microscopy of TUNEL-positive (red) microglia (green) inside grafts at 2 and 4 weeks after injection in *Cx3cr1*^+/−^/5xFAD transgenic mice. Scale bars, 50 μm in the overview and 15 μm in the inset. **f**, Percentage of TUNEL-positive microglia inside grafts. Each symbol represents one graft from *n* = 9 mice per group. Data are presented as mean (± s.e.m.). Significant differences were determined using the two-tailed Mann–Whitney test. **g**, Confocal microscopy and Imaris-3D reconstruction of microglia outside (left) or inside (right) the graft. Scale bars, 20 μm in the confocal acquisition and 10 μm in the Imaris reconstruction. For 3D reconstruction, we considered two regions inside and outside grafts in *n* = 2 mice per group. **h**, Pearson correlation between the number of microglia-contacting plaques and plaque size inside (*r* = 0.53, *P* < 0.0001) and outside (*r* = 0.80, *P* < 0.0001) the grafts. Each symbol represents one compact plaque from *n* = 7 *Cx3cr1*^+/−^/5xFAD mice. **i**, Heat map of the three top differentially regulated genes (**P < 0.01) in microglia FACS-sorted from either isolated cortical grafts or cortical regions outside the transplant that were used as controls. Differentially expressed genes were determined using the limma-voom package in R. Source data

**Extended Data Fig. 1:**
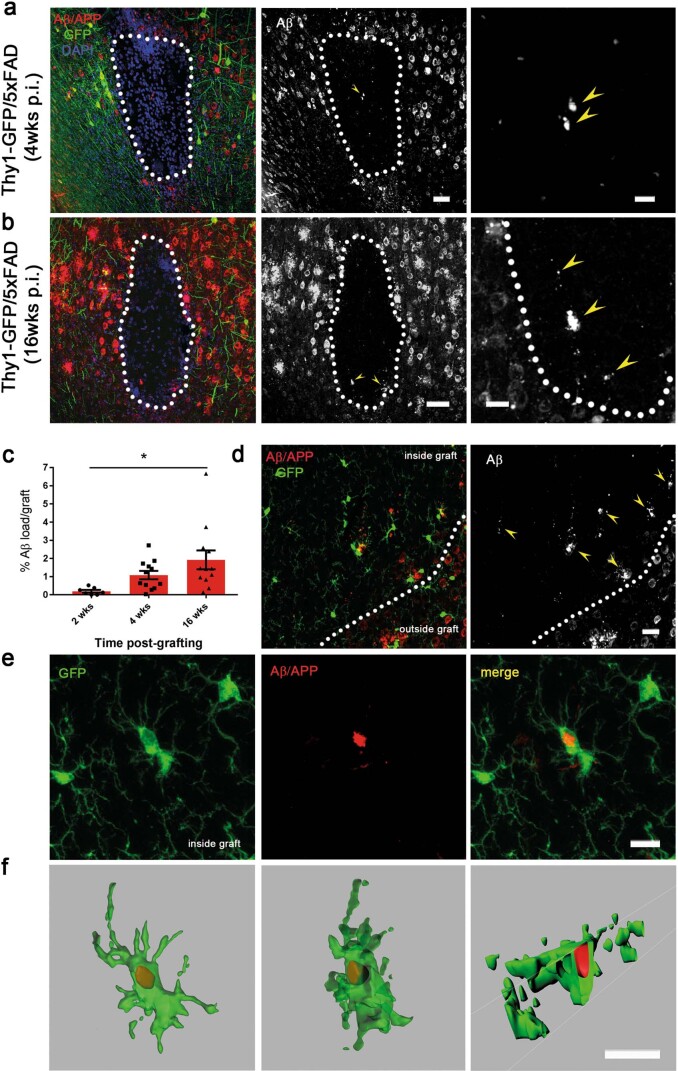
Aβ deposits in WT grafts. (**a**,**b**) Fluorescence microscopy of grafts in Thy1-GFP/5xFAD recipient mice at (a) 4 weeks p.i. and (b) 16 weeks p.i. immunostained for Aβ (6E10, red and white; yellow arrowheads indicate the amyloid deposition) and DAPI (blue). Scale bar, 50 μm in the overview (a) and (b), 20 μm in the higher magnification (a), 25 μm in the higher magnification (b). n = 3 mice per incubation time. (**c**) Histogram showing the percentage of Aβ accumulation inside the grafts of Cx3cr1^+/−^/5xFAD recipient mice at 2, 4 and 16 weeks p.i.. Each symbol represents one graft from n = 4 (2 weeks p.i.), n = 9 (4 weeks p.i.) and n = 8 (16 weeks p.i.) mice. Data are presented as mean (± s.e.m.). Significant differences were determined by one-way ANOVA followed by Tukey’s multiple comparison test, *F*(2, 28) = 4.412 (*P = 0.0175). (**d**-**f**) Representative confocal microscopy showing (d) Aβ localization (6E10, red) in the graft and (**e**) high magnification acquisition (f) and Imaris-3D reconstruction of microglia (GFP, green) containing Aβ (red). Scale bars, 50 μm in (d), 10 μm in (e) and (f). n = 3 mice. Source data

**Extended Data Fig. 2:**
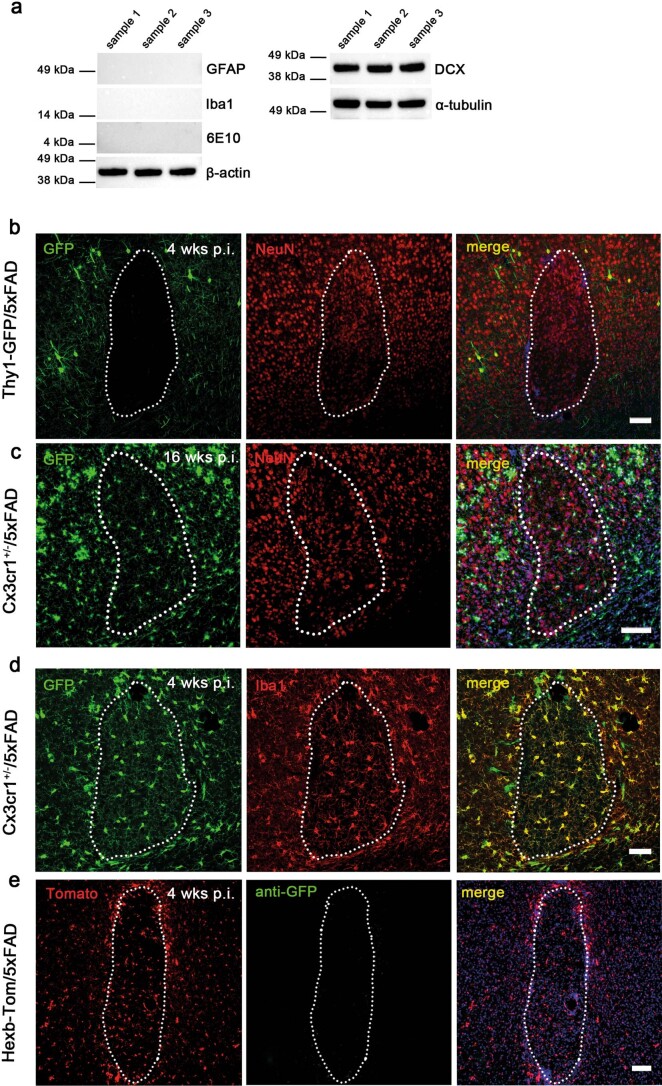
Cellular composition of cortical WT grafts. **(a**) Immunoblot analysis of cortical cell suspension that were used for grafting experiments. For the blot in the left panel, every protein was detected on separate membranes and only the loading control from the GFAP gel is shown. For uncropped gels with corresponding loading controls see Source Data Extended Data Fig. 2. n = 3 independent cell suspensions. **(b)** Representative confocal image of a graft in Thy1-GFP/5xFAD recipient mouse immunostained with NeuN (red) and DAPI (blue). n = 1 mouse. (**c**) Representative microscopy of a graft at 16 weeks p.i. of a Cx3cr1^+/−^/5xFAD recipient mouse immunostained with NeuN (red) and DAPI (blue). n = 1 mouse. (**d**) Confocal image of a graft in Cx3cr1^+/−^/5xFAD recipient mouse immunostained with Iba1 (red). n = 3 mice. (**e**) Confocal image of a graft in Hexb-tom/5xFAD recipient mouse transplanted with neurons from Cx3cr1-GFP embryos and counterstained with DAPI (blue). Scale bars 100 μm in (b), (c) and (e), 50 μm in (d). n = 4 mice. Source data

**Extended Data Fig. 3:**
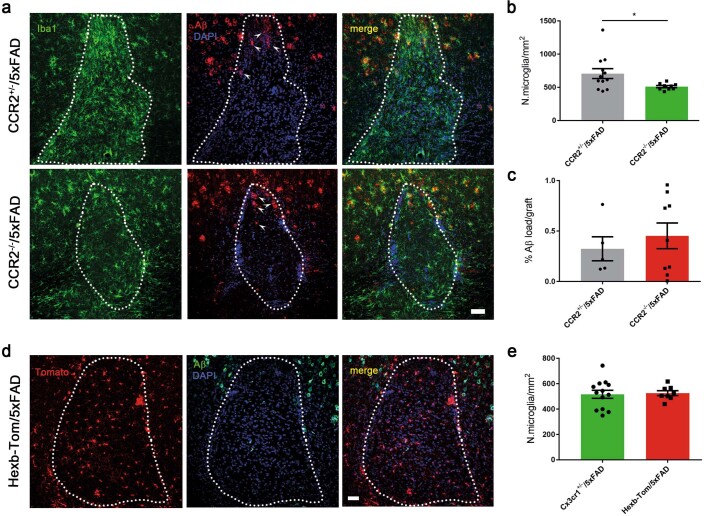
Resident microglia influence Aβ accumulation within the grafts. (**a**) Fluorescence microscopy of a graft in CCR2^+/−^/5xFAD (top) and CCR2^−/−^/5xFAD (bottom) mice immunostained for Aβ (6E10, red; white arrowheads indicate the Aβ), Iba1 (green) and DAPI (blue). Scale bar, 50 μm. (**b** and **c**) Graphs show mean (± s.e.m.) of (**b**) microglia density and (c) percentage of Aβ inside grafts in the two groups of mice. Each symbol represents one graft from n = 7 CCR2^+/−^/5xFAD and n = 4 CCR2^−/−^/5xFAD mice. Significant differences were determined by the Mann–Whitney test (*P = 0.04). (**d**) Fluorescence microscopy of a graft in Hexb-tom/5xFAD recipient immunostained for Aβ (6E10, green), and DAPI (blue). Scale bar, 50 μm. (**e**) Histogram compares mean (± s.e.m.) of microglia density in grafts of Cx3cr1^+/−^/5xFAD and Hexb-tom/5xFAD recipient mice. Each symbol represents one graft from n = 8 Cx3cr1^+/−^/5xFAD and n = 4 Hexb-tom/5xFAD. Significant differences were determined by the two-tailed Mann–Whitney test. Source data

In this study, we transplanted embryonic neuronal cells from WT mice into the neocortex of young, pre-depositing 5xFAD transgenic mice, confirming transplant integration into the host tissue as well as the survival of the grafts over several months (Fig. 1a,b and Extended Data Fig. 1a,b)^13^. As soon as 4 weeks after transplantation, Aβ plaques were present within WT grafts, and they increased over time (Fig. 1a and Extended Data Fig. 1a–c, yellow arrowheads). We first hypothesized that APP/Aβ is anterogradely transported from the host transgenic neurons into the WT grafts, and we used *Thy1-GFP*/5xFAD mice as recipients to visualize the alignment of processes from the host mouse. Although we found a few fibers that crossed the host–graft boundary, most of the green fluorescent protein (GFP)-positive cells did not penetrate into the WT graft (Fig. 1b and Extended Data Fig. 1a,b), indicating that axonal transport is not likely to be involved in the propagation of Aβ pathology into WT grafts. The greatest amyloid accumulation manifested along the border, an area known to have a slightly higher microglia density. Therefore, we investigated whether, indeed, host microglia are able to invade the WT graft by using *Cx3cr1*^+/−^/5xFAD mice. By 2 weeks after transplantation, massive invasion of CX3CR1-positive cells into the graft was evident, probably owing to injury or the placement of the graft itself, with the highest number of cells at the edges (Fig. 1c). This microglia accumulation ceased over time (Fig. 1d), although there was no prominent cell death (Fig. 1e,f). We observed many small Aβ particles within the cells or intimately associated with microglia (Extended Data Fig. 1d–f). Next, we characterized the cellular composition of the graft and confirmed the presence of doublecortin (DCX) in cellular samples, whereas we did not observe any signal for GFAP or IBA1 (Extended Data Fig. 2a). Furthermore, we detected NeuN-positive neurons at 4 and 16 weeks after transplantation as well as CX3CR1-positive microglia that were all double-labeled with IBA1 (Extended Data Fig. 2b–d). To prove that the microglia were indeed host-derived and not donor-derived, we transplanted embryonic cell suspensions from *Cx3Cr1-GFP* mice into *Hexb*^*tdTomato*^ recipients that express tdTomato exclusively in resident microglia in the central nervous system^16^. No GFP-positive donor-derived cells could be detected in the graft, excluding microglia contamination from the donors (Extended Data Fig. 2e). To rule out a contribution of infiltrating monocytes from the periphery, we used *Ccr2*^−/−^/5xFAD mice as recipients, which had similar Aβ load within the grafts when compared to *Ccr2*^+/−^/5xFAD mice, despite having significantly fewer IBA1-positive cells (Extended Data Fig. 3a–c). Likewise, the number of microglia within grafts placed either in *Cx3cr1*^+/−^/5xFAD or *Hexb*^*tdTomato*^/5xFAD recipients was similar (Extended Data Fig. 3d,e), confirming that resident microglia rather than peripheral monocytes from the hosts were infiltrating the grafts. Microglia within the grafts displayed the typical arborization with fine delineated processes similar to non-plaque-associated microglia outside the grafts (Fig. 1g), and the number of microglia surrounding amyloid plaques increased with plaque size independent of their graft or host association (Fig. 1h). By performing bulk RNA sequencing (RNA-seq) of fluorescence-activated cell-sorted (FACS) microglia derived from within or outside the grafts, we identified only three differentially expressed genes out of 15,548 genes (Fig. 1i and Supplementary Fig. 1), indicating that host microglia maintain their transcriptome after migration to the graft and reaffirming that microglia in both compartments are similar.

**Fig. 2:**
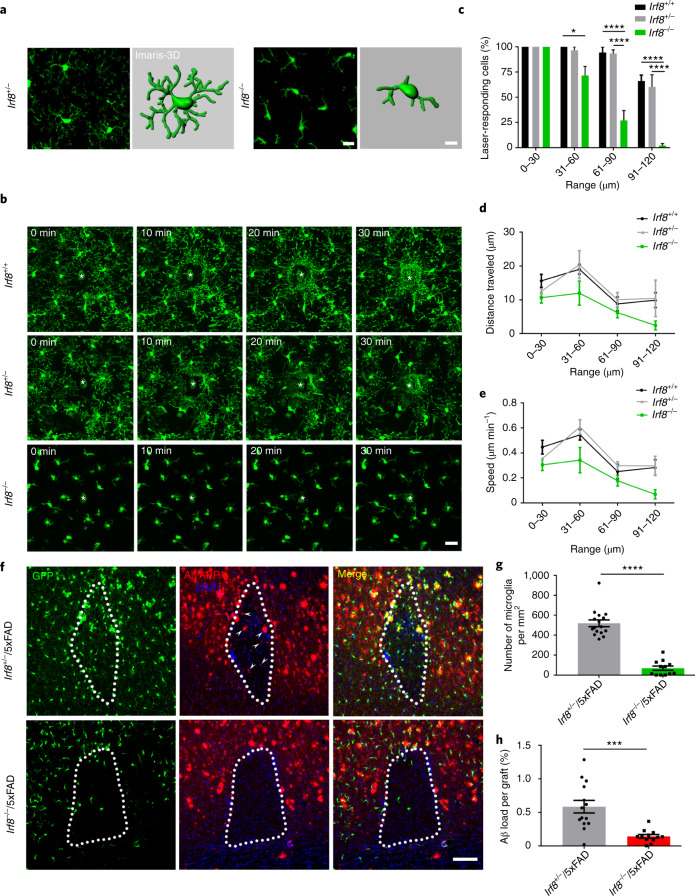
Decreased Aβ deposition inside grafts in *Irf8*^−/−^/5xFAD recipient mice. **a**, Representative confocal images (left) and corresponding Imaris-3D reconstruction (right) of microglia in the cortex of *Irf8*^+/−^ and *Irf8*^−/−^ mice. Scale bars, 15 μm in the left image and 10 μm in the 3D reconstruction on the right. For 3D reconstruction, two cells were considered in *n* = 2 mice per group. **b**–**e**, In vivo two-photon analysis of microglia process motility between 0 and 30 min after laser lesion ablation in *Irf8*^+/+^, *Irf8*^+/−^ and *Irf8*^−/−^ mice. Scale bar, 20 μm. Quantification of percentage of laser-responding cells (**c**), mean distance traveled (**d**) and speed of microglia processes (**e**) based on their distance from the injury site. Data are presented as mean (± s.e.m.). Significant differences were determined using two-way ANOVA followed by Sidak’s multiple comparison test (in **c**, ****P < 0.0001 and *P = 0.030), Tukey’s multiple comparison test *F*_6,46_ = 0.8661 (in **d**, range 31–60 μm: *P = 0.044 *Irf8*^+/+^ versus *Irf8*^−/−^ and **P = 0.008 *Irf8*^+/−^ versus *Irf8*^−/−^; range 91–120 μm: *P* = 0.054 *Irf8*^+/+^ versus *Irf8*^−/−^ and *P = 0.0199 *Irf8*^+/−^ versus *Irf8*^−/−^) and Tukey’s multiple comparison test *F*_6,46_ = 0.9551 (in **e**, range 31–60 μm: *P = 0.0484 *Irf8*^+/+^ versus *Irf8*^−/−^ and **P = 0.0039 *Irf8*^+/−^ versus *Irf8*^−/−^; range 91–120 μm: *P* = 0.0584 *Irf8*^+/+^ versus *Irf8*^−/−^ and *P = 0.0235 *Irf8*^+/−^ versus *Irf8*^−/−^). Data are from *n* = 4 *Irf8*^+/+^, *n* = 5 *Irf8*^+/−^ and *n* = 6 *Irf8*^−/−^ mice. **f**, Grafts in *Irf8*^+/−^/5xFAD and *Irf8*^−/−^/5xFAD recipient mice immunostained for Aβ (6E10, red; white arrowheads indicate Aβ depositions) and DAPI (blue). Scale bar, 100 μm. **g**,**h**, Graphs show mean (± s.e.m.) microglia density (**g**) and percentage of Aβ inside the grafts (**h**) in the two groups of mice. Each symbol represents one graft from *n* = 7 *Irf8*^+/−^/5xFAD and *n* = 6 *Irf8*^−/−^/5xFAD mice. Significant differences were determined using the two-tailed Mann–Whitney test (****P < 0.0001 in **g** and ***P = 0.001 in **h**). Source data

**Fig. 3:**
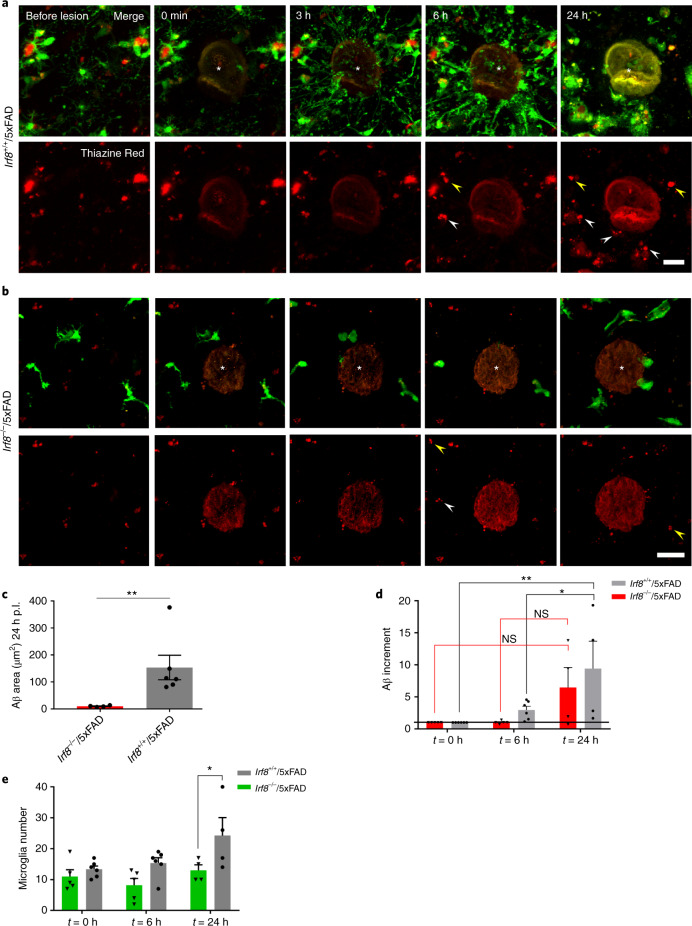
In vivo two-photon imaging of Aβ-containing microglia movement after laser lesion. **a**,**b**, In vivo two-photon imaging of Aβ-containing microglial responses at different time points after laser-induced tissue injury in *Irf8*^+/+^/*Cx3cr1*^+/−^/5xFAD mice (**a**) and *Irf8*^−/−^/*Cx3cr1*^+/−^/5xFAD mice (**b**). The top panels show merged images of GFP (green) and Thiazine Red (red) signal; the bottom panel shows only the Thiazine Red signal; asterisks indicate laser ablations; white arrowheads depict internalized Aβ moving with time; and yellow arrowheads indicate amyloid material associated with small microglia debris or no microglia association at all (shown only in the red channel). Scale bars, 50 μm. **c**–**e**, Graphs show mean (± s.e.m.) of Aβ accumulation at the lesion site 24 h post lesion (p.l.) in both groups of mice (**c**), relative increment of Aβ at the lesion site at 0, 6 and 24 h p.l. (**d**), and microglia accumulation at 0, 6 and 24 h p.l (**e**). Each symbol represents one laser lesion from *n* = 6 *Irf8*^+/+^/*Cx3cr1*^+/−^/5xFAD mice and *n* = 5 *Irf8*^−/−^/*Cx3cr1*^+/−^/5xFAD mice. Significant differences were determined using either the two-tailed Mann–Whitney test (**P = 0.0095 in **c**) or two-way ANOVA with Sidak’s multiple comparison test (*P = 0.0436 and **P = 0.0068 in **d** and *P = 0.0282 in **e**). NS, not significant. Source data

**Extended Data Fig. 4:**
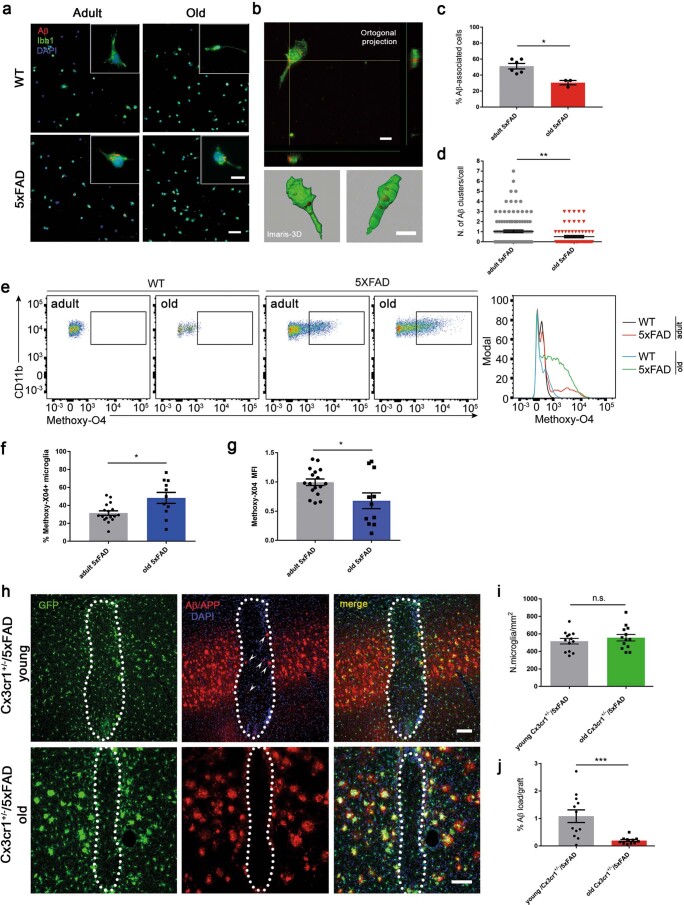
Less Aβ deposition inside grafts of adult recipient mice. (**a** and **b**) Confocal microscopy of microglia cells in vitro isolated from adult (WT and 5xFAD) or old (WT and 5xFAD) mice immunostained for Aβ (6E10, red), Iba1 (green) and DAPI (blue). Scale bar 50 μm in the overview and 10 μm in the inset. (**b**) Confocal orthogonal projection (top) and Imaris-3D reconstruction (bottom) of a single microglia cell containing Aβ material. Scale bars, 10 μm. (**c** and **d**) Graph shows mean (± s.e.m.) of Aβ-associated microglia (**c**) in cells isolated from adult and old 5xFAD mice. Each symbol represents data from one mouse, n = 6 adult 5xFAD and n = 3 old 5xFAD mice. Significant differences were determined by the two-tailed Mann–Whitney test (*P = 0.023). (**d**) Number of Aβ clusters inside each microglia cell from adult and old 5xFAD mice. Each symbol represents one cell. From n = 5 adult 5xFAD and n = 3 old 5xFAD mice. Significant differences were determined by the two-tailed Mann–Whitney test (**P = 0.0038). (**e-g**) Methoxy-XO4 FACS analysis of microglia isolated from adult and old 5xFAD mice. (**e**) Representative FACS dot-plots (left) and cytometry graph (right) for WT, adult 5xFAD and old 5xFAD mice. Quantification of the (**f**) percentage (%) and (**g**) mean fluorescence intensity (MFI) of methoxy-XO4 positive CD11b^+^CD45^low^ hippocampal microglial cells by flow cytometry isolated from the two 5xFAD groups 3 hours after methoxy-XO4 injection. Each symbol represents data from one mouse, with n = 17 adult 5xFAD and n = 11 old 5xFAD mice. Data are presented as mean (± s.e.m.). Significant differences were determined by the two-tailed Mann–Whitney test (*P = 0.022 in f; *P = 0.045 in g). (**h**) Fluorescence microscopy of grafts in young pre-depositing (top) and old (bottom) Cx3cr1^+/−^/5xFAD mice immunostained for Aβ (6E10, red; white arrowheads indicate Aβ deposition) and DAPI (blue). Scale bar, 100 μm. (**i** and **j**) Graphs show mean (± s.e.m.) of (**i**) microglia density and (**j**) percentage of Aβ inside grafts in the two groups of mice. Each symbol represents one graft from n = 9 young Cx3cr1^+/−^/5xFAD and n = 7 old Cx3cr1^+/−^/5xFAD mice. Significant differences were determined by the two-tailed Mann–Whitney test (*P* = 0.544 in i; ***P = 0.0005 in j). Source data

**Extended Data Fig. 5:**
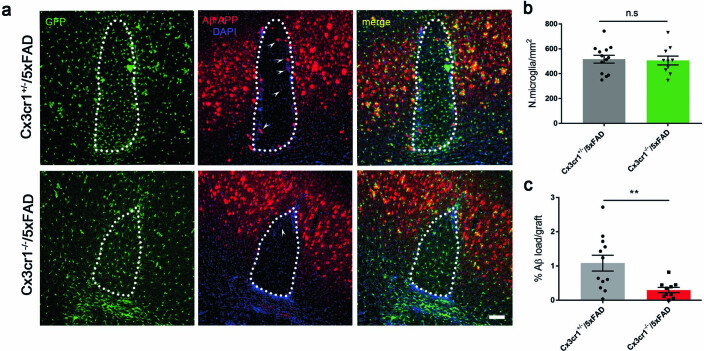
Reduced Aβ deposition inside grafts of Cx3cr1^−/−^/5xFAD recipient mice. (**a)** Fluorescence microscopy of a graft in Cx3cr1^+/−^/5xFAD (top) and Cx3cr1^−/−^/5xFAD (bottom) mice immunostained for Aβ (6E10, red; white arrowheads indicate Aβ), and DAPI (blue) at 4 weeks p.i. Scale bar, 100 μm. (**b** and **c**) Graphs show mean (± s.e.m.) of (**b**) microglia density and (**c**) percentage of Aβ inside the grafts. Each symbol represents one graft from n = 9 Cx3cr1^+/−^/5xFAD and n = 7 Cx3cr1^−/−^/5xFAD mice. Significant differences were determined by the two-tailed Mann–Whitney test (**P = 0.009). Source data

**Extended Data Fig. 6:**
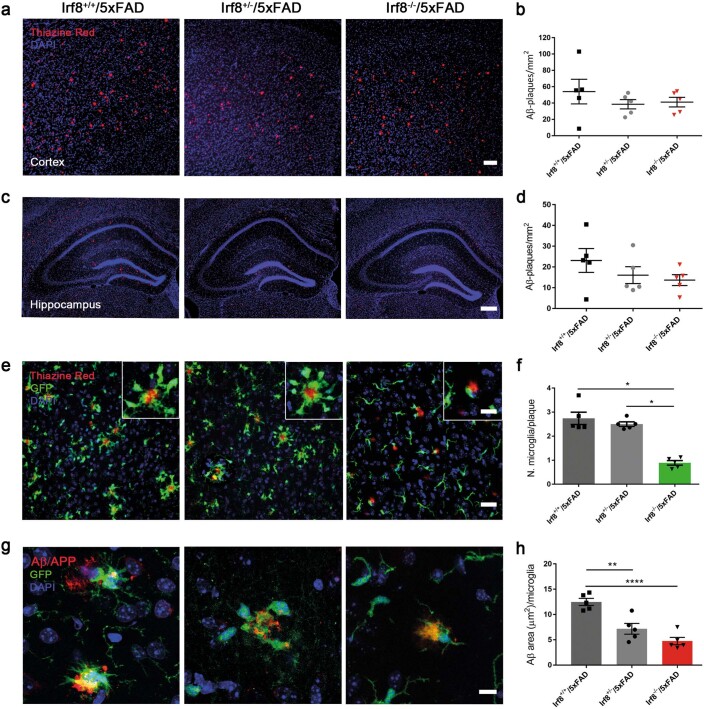
Quantification of Aβ-plaques in Irf8^−/−^/Cx3cr1^+/−^/5xFAD mice. **(a,c)** Fluorescence microscopy of Irf8^+/+^/Cx3cr1^+/−^/5xFAD (left), Irf8^+/−^/Cx3cr1^+/−^**/**5xFAD (middle) and Irf8^−/−^/Cx3cr1^+/−^**/**5xFAD (right) cortices (**a**) and hippocampi (**c**) stained with Thiazine Red (red) and DAPI (blue). Scale bar, 100 μm in (**a**); 300 μm in (**c**). (**b,d**) Graphs show the mean (± s.e.m.) of Thiazine Red positive Aβ plaques in the three groups of mice in the cortex (**b**) and hippocampus (**d**). Each symbol represents one mouse from n = 5 mice per group. Significant differences were determined by one-way ANOVA followed by Tukey’s multiple comparison test. (**e**) Confocal microscopy of Irf8^+/+^/Cx3cr1^+/−^**/**5xFAD (left), Irf8^+/−^/Cx3cr1^+/−^**/**5xFAD (middle) and Irf8^−/−^/Cx3cr1^+/−^**/**5xFAD (right) cortices stained with Thiazine Red (red) and DAPI (blue). Scale bar, 30 μm; inset, 10 μm. (**f**) Graph shows the mean number (± s.e.m.) of microglia per plaque in the three groups. Each symbol represents one mouse from n = 5 mice per genotype. Significant differences were determined by Kruskal-Wallis test followed by Dunn’s multiple comparisons test, (*P = 0.014; *P = 0.04). (**g**) Confocal microscopy of Irf8^+/+^/Cx3cr1^+/−^**/**5xFAD (left), Irf8^+/−^/Cx3cr1^+/−^**/**5xFAD (middle) and Irf8^−/−^/Cx3cr1^+/−^**/**5xFAD (right) cortices immunolabeled with 6E10 antibody (red) and DAPI (blue). Scale bar, 10 μm. (**h**) Graph shows the mean (± s.e.m.) of Aβ internalized by microglia in the three groups of mice. Each symbol represents one mouse from n = 5 mice per group. Significant differences were determined by one-way ANOVA followed by Tukey’s multiple comparison test, *F*(2, 12) = 21.8 (**P = 0.0022; ****P < 0.0001). Source data

**Extended Data Fig. 7:**
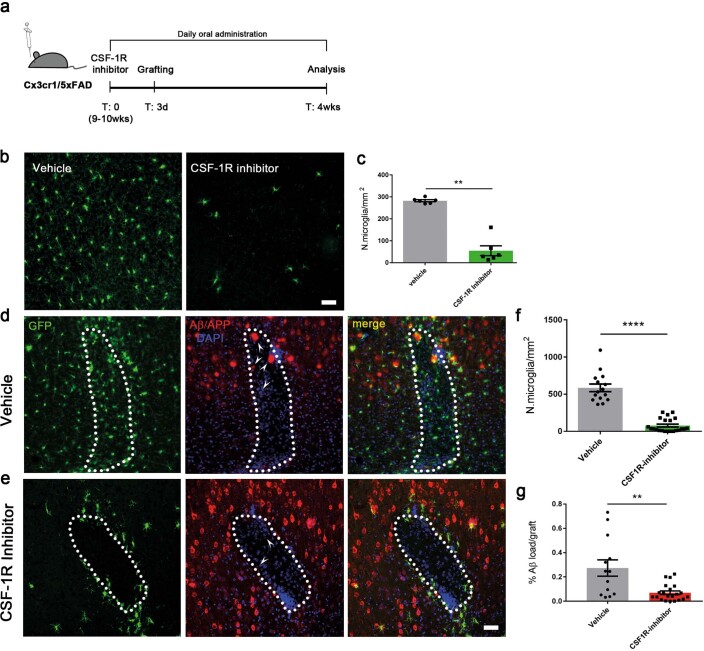
Reduced Aβ deposition inside grafts of microglia-depleted Cx3cr1^+/−^/5xFAD mice. (**a**) Experimental setup for the elimination of microglia and transplantation procedure in Cx3cr1^+/−^/5xFAD mice. (**b**) Fluorescence microscopy of Cx3cr1^+/−^/5xFAD cortex treated with vehicle (left) or CSF-1R inhibitor (right). Scale bar, 50 μm. (**c**) Quantification shows mean (± s.e.m.) of cortical microglia density after treatment. Each symbol represents one mouse of n = 6 mice per group. Significant differences were determined by the two-tailed Mann–Whitney test (**P = 0.0022). (**d-e**) Fluorescence microscopy of a graft in Cx3cr1^+/−^/5xFAD mice treated for 30 days with (**d**) CSF-1R vehicle or (**e**) CSF-1R inhibitor and immunostained for Aβ (6E10, red; white arrowheads indicate Aβ), and DAPI (blue). Scale bar, 50 μm. (**f** and **g**) Graphs show mean (± s.e.m.) of (**f**) microglia density and (**g**) percentage of Aβ inside the graft in the two groups of mice. Each symbol represents one graft from n = 8 mice per group. Significant differences were determined by the two-tailed Mann–Whitney test (****P < 0.0001; **P = 0.0031). Source data

**Extended Data Fig. 8:**
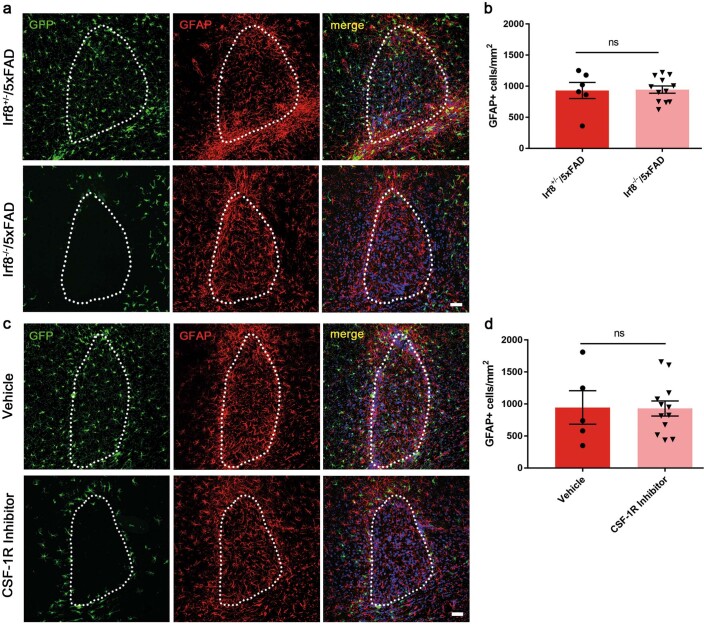
Astrocytes accumulate in grafts of Irf8^−/−^/Cx3cr1^+/−^/5xFAD and microglia depleted Cx3cr1^+/−^/5xFAD mice. (**a,c)** Confocal image of a graft in **(a)** Irf8^+/−^/Cx3cr1^+/−^**/**5xFAD (top), Irf8^−/−^/Cx3cr1^+/−^**/**5xFAD (bottom) and **(c)** Cx3cr1^+/−^/5xFAD mouse treated with vehicle (top) or with CSF-1R inhibitor (bottom) immunostained with an antibody against GFAP (red) and DAPI (blue). Scale bar, 50 μm. (**b,d**) Graphs show mean density (± s.e.m.) of GFAP-positive astrocytes in (**b**) Irf8/Cx3cr1^+/−^**/**5xFAD and in (**d**) microglia depleted mice. Each symbol represents one graft from n = 4 mice per genotype. Significant differences were determined by the two-tailed Mann–Whitney test. Source data

**Extended Data Fig. 9:**
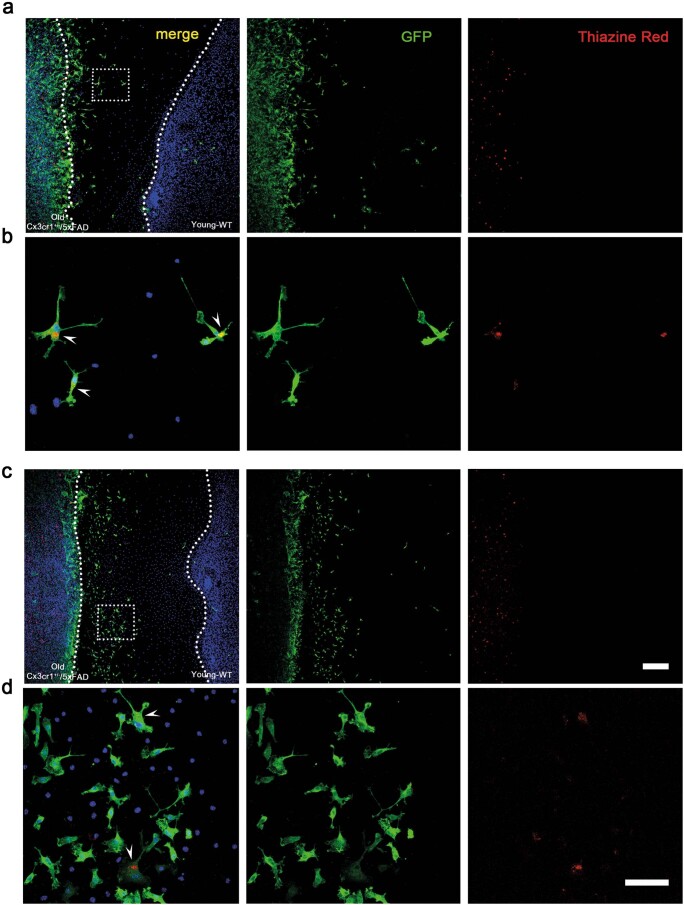
Microglia migrate and transport Aβ from the old Cx3cr1^+/−^/5xFAD to the young WT brain slices in the co-culture model. (**a-d**) Confocal images of co-cultures of organotypic brain slices from aged Cx3cr1^+/−^/5xFAD and postnatal WT mice. Slices were immunolabelled with GFP (green), Thiazine Red (red) and Hoechst (blue). (**a,c**) Overview images of two representative co-cultures. Boxed regions in (**a, c**) are shown with a higher magnification in (**b, d**) and depict migrating 5xFAD microglia (GFP^+^) carrying Aβ (Τhiazine Red^+^) from the aged AD tissue towards the young WT tissue. Scale bars, 200 µm (**a, c**), 50 µm (**b, d**). n = 9 co-cultured brain slices from n = 2 mice per genotype.

The fact that microglia are phagocytic cells and, as shown, invade the graft prompted us to speculate that microglia take up Aβ from the recipient tissue and invade the WT graft, where Aβ finally deposits. Manipulating microglial function should, therefore, alter this process. Because the capacity of microglia to phagocytose Aβ is compromised in the aging brain and AD^17–20^, we performed in vitro experiments to compare the amount of Aβ incorporated by microglia of adult and old 5xFAD mice (Extended Data Fig. 4a). Both the percentage of Aβ-containing microglia and the number of Aβ puncta per cell were significantly decreased in old mice (Extended Data Fig. 4b–d). Next, we analyzed the phagocytic capacity of microglia in vivo after administration of methoxy‐XO4. Flow cytometric analysis of microglia from adult and old WT and 5xFAD mice (Extended Data Fig. 4e and Supplementary Fig. 2) showed a significantly higher percentage of methoxy-XO4-positive microglia in old compared to adult animals (Extended Data Fig. 4f), whereas mean fluorescence intensity (MFI) of methoxy-XO4 was significantly decreased in microglia from old 5xFAD mice (Extended Data Fig. 4g). These data confirmed a decline in microglial phagocytosis of Aβ in old 5xFAD mice. Finally, we used young or old *Cx3cr1*^+/−^/5xFAD mice as recipients and compared the amyloid load inside the grafts 4 weeks later. The amount of Aβ within the grafts of old mice was strongly reduced, even though microglia density was equivalent (Extended Data Fig. 4h–j), suggesting that compromised phagocytosis by aged microglia in the transgenic host tissue subsequently lead to a decrease in amyloid deposition in WT grafts. We validated our hypothesis further by using *Cx3cr1*-deficient 5xFAD transgenic mice as recipients. In accordance with the higher Aβ clearance propensity in mice lacking CX3CR1 (ref. ^21^), the amyloid load was again significantly reduced in the grafts, although the number of microglia in both genotypes was similar (Extended Data Fig. 5a–c). Next we took advantage of *Irf8*^−/−^/*Cx3cr1*^+/−^ transgenic mice, which are characterized by microglia with reduced and swollen processes^22,23^ and reduced motility^24^. Indeed, IRF8-deficient microglia exhibited morphological alterations, such as reduced and shorter branches (Fig. 2a)^22^. Under steady-state conditions, microglial process motility in *Irf8*^−/−^ mice was strongly impaired (Supplementary Videos 1 and 2). Furthermore, focal-laser-induced damage followed by time lapse in vivo two-photon imaging revealed a diminished microglia response to the lesion in *Irf8*^−/−^ mice (Supplementary Videos 3 and 4 and Fig. 2b). Quantification of responding cells based on their distance from the injury site identified significantly fewer responding cells in *Irf8*^−/−^ mice than in control groups (Fig. 2c). In contrast to the cell bodies, which remained at their original positions^25,26^, the speed of microglial processes and the distance to the injury declined markedly in these mice (Fig. 2d,e). We then generated *Irf8*^−/−^/*Cx3cr1*^+/−^/5xFAD mice, which had similar numbers of Aβ plaques (Extended Data Fig. 6a–d) with significantly less microglial clustering around Aβ plaques and a smaller amount of internalized Aβ (Extended Data Fig. 6e–h). Notably, microglia failed to migrate into the WT grafts, and, thus, the number of microglia that entered the grafts was substantially reduced, resulting in a reduction in amyloid deposits in the grafts (Fig. 2f–h), further corroborating our hypothesis. Strikingly, we also found evidence for microglia propagating Aβ into WT tissue by depleting microglia using the colony-stimulating factor 1 receptor (CSF-1R) inhibitor BLZ945 in *Cx3cr1*^+/−^/5xFAD recipient mice (Extended Data Fig. 7a). We obtained an approximately 80% cell reduction in the cortex of mice treated with the CSF-1R inhibitor as well as within the grafts (Extended Data Fig. 7b–f), resulting, again, in significantly fewer Aβ deposits within the grafts (Extended Data Fig. 7e,g). Because the elimination of microglia did not completely prevent Aβ plaque formation within the grafts, we cannot rule out the possibility that additional factors (for example, diffusion) at least partially contribute to this propagation process. Neither CSF-1R inhibitor treatment nor IRF8 deficiency had an effect on the presence of astrocytes within the grafts, despite lowered amyloid content (Extended Data Fig. 8a–d), indicating that astrocytes might play only a minor role in amyloid propagation, at least in our grafting model system. To finally demonstrate the active involvement of microglia in Aβ propagation into grafts, we performed repetitive two-photon in vivo imaging of *Irf8*^+/+^/*Cx3cr1*^+/−^/5xFAD mice in comparison to *Irf8*^−/−^/*Cx3cr1*^+/−^/5xFAD mice and examined microglial motility associated with Aβ transport in response to a laser-induced focal tissue injury. Microglia moved to the lesion site, where Aβ plaques started to build up concomitantly (Fig. 3a,c,d,e) owing to microglial transport of Aβ toward the lesion site (Supplementary Videos 5 and 6). By contrast, movement of Aβ-laden microglia toward the lesion site was not evident in *Irf8*^−/−^/*Cx3cr1*^+/−^/5xFAD mice (Fig. 3b). We further validated our findings in the absence of tissue injury by co-culturing young brain slices from WT animals with old *Cx3cr1*^+/−^/5xFAD brain slices, as recently reported^20^. We detected at 14 days in vitro many GFP-positive microglia migrating toward the WT tissue, with fewer cells reaching the edges of the young tissue and only some that migrated deeper into the tissue (Extended Data Fig. 9a,c). Upon closer inspection, we noticed Aβ-containing microglia clearly migrating from the old *Cx3cr1*^+/−^/5xFAD brain slice to the young WT slice (Extended Data Fig. 9b,d), confirming the notion that Aβ is transported by microglia into non-diseased brain tissue.

Together, these findings highlight the importance of microglia for the propagation of Aβ pathology into WT grafts by acting as Aβ carriers that migrate toward previously unaffected brain tissue. By using neural grafting experiments in 5xFAD mice with altered microglial function, we propose that the formation of Aβ plaques in grafted, unaffected tissue is, at least to some extent, dependent on host microglia functionality (Supplementary Fig. 3). Although our findings need to be repeated in a physiologically more relevant context, one can envision that physical trauma, traumatic brain injury, stroke, tumor or neurodegeneration might become the trigger for microglia migration and transport of Aβ. We conclude that targeting microglia function might provide an opportunity to interfere with the propagation of Aβ.

## Methods

### Animals

To visualize neuronal structures, *Thy1-eGFP* mice^27^ were crossbred with 5xFAD transgenic mice co-expressing human APP^K670N/M671L(Sw)+I716V(Fl)+V717I(Lo)^ and PS1^M146L+L286V^ under the control of the neuron-specific *Thy1* promoter^28^. To visualize green fluorescence in microglia, we intercrossed *Cx3cr1*^*GFP/wt*^/5xFAD (here reported as *Cx3cr1*^+/−^/5xFAD)^29^ mice to obtain *Cx3cr1*^+/−^/5xFAD or *Cx3cr1*^−/−^/5xFAD; to visualize red fluorescence in microglia, we crossed *Hexb*^*tdTomato*^ with 5xFAD mice and obtained homozygous *Hexb*^*tdTomato*^/5xFAD mice. We backcrossed 5xFAD with *Irf8*^−/−^/*Cx3cr1*^*GFP/GFP*^ mice (courtesy of Clemens Lange) to obtain *Irf8*^+/+^/*Cx3cr1*^+/−^ /5xFAD, *Irf8*^+/−^/*Cx3cr1*^+/−^/5xFAD or *Irf8*^−/−^/*Cx3cr1*^+/−^/5xFAD mice. The *Hexb*^*tdTomato*^ and *Ccr2*^−/−^/5xFAD transgenic mice were kindly provided by Marco Prinz. All mice were on a C57BL/6 background. For this study, only female mice were used, to minimize variability and reduce sample size. Animals were group-housed under specific pathogen-free conditions and kept under a 12-h light–dark cycle with food and water ad libitum at a temperature of 22 °C and 73% humidity. For all experiments, mice were randomly allocated into each experimental group. All animal experiments were carried out in accordance with the policies of the State of Baden-Württemberg under license number G16-100.

### Preparation of cortical cell suspensions for intracerebral grafting

Primary cortical neurons were isolated from C57BL/6 WT mice at embryonic day 16–17. Cortices were dissected on ice, trypsinized for 10 min with 0.05% Trypsin-EDTA (Gibco) at 37 °C and washed three times in HBSS (Gibco). Cells were triturated in DMEM (Gibco) by pipetting up and down until the suspension was homogenous and maintained on ice until the injection.

### Intracerebral grafting

Mice were anesthetized via intraperitoneal (i.p.) injection with a mixture of ketamine (100 mg kg^−1^ body weight) and xylazine (10 mg kg^−1^ body weight) dissolved in saline. For bilateral stereotactic transplantation of neuronal cell suspensions, a Hamilton syringe was placed into the cortex (AP +1.8 mm, L ±1.5 mm, DV −1.3 mm) of 9–10-week-old (young adult) or 8–11-month-old (adult) mice. Animals were injected with 3 µl (approximately 20,000 cells) per hemisphere at an injection speed of 1 µl min^−1^. After each injection, the needle was kept in place for 1 min before it was slowly withdrawn. The surgical site was cleaned with sterile saline and the incision sutured. Mice were monitored until recovery from anesthesia and followed for 2, 4 or 16 weeks.

### Isolation of primary microglia for in vitro culture

The whole brain from adult (20–30-week-old) or old (50–60-week-old) mice was isolated after transcardial perfusion with 1× PBS, and a single-cell suspension was prepared. The homogenate was filtered through a cell strainer (70 µm) and separated by 37% Percoll gradient centrifugation at 400*g* for 30 min at 4 °C. The myeloid-containing phase was collected and washed once with HBSS. The cells were resuspended in culture medium (DMEM/F-12, 1× penicillin–streptomycin, 10% FCS) and plated onto poly-lysinated coverslips for 24 h at 37 °C and 5% CO_2_. The cells were quickly washed with 1× PBS and fixed with 4% paraformaldehyde (PFA) for 15 min. Microglia were isolated from 3–6 animals per group.

### Histology and immunocytochemistry

Mice were overdosed with an i.p. injection of ketamine and xylazine and transcardially perfused with 10 ml of ice-cold PBS followed by 10 ml of ice-cold 4% PFA (ROTI-Histofix, Carl Roth) in PBS. Brains were isolated and post-fixed in 4% PFA for 24 h at 4 °C, followed by incubation in 30% sucrose (in PBS, pH 7.5) for 48 h. Frozen brains were cut into 25 µm-thick coronal sections on a sliding microtome (SM2000R, Leica Biosystems) and collected in 15% glycerol dissolved in PBS. Sections were incubated overnight at 4 °C with the following antibodies diluted in 1× PBS containing 5% normal goat serum (NGS) and 0.5% Triton X-100: anti-IBA1 (rabbit, 1:500, Abcam, ab178846), anti-GFAP (mouse, 1:500, Sigma-Aldrich, GA5), anti-Aβ (mouse, 1:1,000, Covance, 6E10) and anti-NeuN (mouse, 1:500, Abcam). Appropriate secondary antibodies conjugated to Alexa Fluor 488 or 555 (1:1,000) were used before counterstaining with DAPI (1:10,000).

Primary microglia cultures were immunolabeled using anti-Aβ (mouse, 1:1,000, Covance, 6E10) and anti-IBA1 (rabbit, 1:1,000, Wako, 019-19741) diluted in 1× PBS containing 5% NGS overnight at 4 °C and incubated for 1.5 h with secondary antibodies conjugated to Alexa Fluor 488 or 555 (1:1,000) and finally counterstained with DAPI (1:10,000).

### TUNEL apoptosis assay

For the labeling of apoptotic cells inside grafts, the In Situ Cell Death Detection Kit (Roche) was used. This method is based on TUNEL. Sections were mounted on glass slides and dried overnight. The slides were post-fixed in 4% PFA for 10 min and washed for 20 min in PBS, followed by permeabilization in ice-cold 0.5% Triton X-100 and 0.1% sodium citrate for 2 min at 4 °C. The freshly prepared TUNEL reaction mixture was kept in the dark and on ice until use. The slides were incubated with 50 μl of TUNEL reaction mixture in a humid chamber for 1 h at 37 °C. After this incubation step, slides were washed three times for 5 min each in PBS, dried, and covered with mounting medium and a glass coverslip. Slides were kept in the dark at 4 °C until they were used for microscopy. As a positive control, sections were incubated with recombinant DNase I. For double labeling of TUNEL and antibodies, first, antibody labeling was done, followed by staining with the TUNEL kit.

Confocal images of the stained grafts were taken with an Olympus confocal microscope (FluoView FV 1000), and the percentage of TUNEL-positive microglial cells inside grafts was quantified using ImageJ (version 1.52a).

### Microglia counting and assessment of Aβ

Every tenth brain section was immunohistochemically labeled with the 6E10 antibody specific to human Aβ/APP to easily identify the graft, as grafted WT cells are negative for APP, whereas the recipient surrounding tissue has a high content of intracellular APP staining. Fluorescence sections were imaged with a Zeiss fluorescence microscope (Axio Imager M2M) and opened with ImageJ for analysis. Total Aβ load in the graft was determined by calculating the per cent area fraction occupied by Aβ-positive staining in the graft, and the density of microglia inside the graft was counted manually. Each data point represents one graft in the histogram. Data were excluded if intracerebral grafting was unsuccessful or no graft was present.

Microglia depletion efficiency was determined by counting the number of GFP-positive microglia in the cortex of the CSF-1R- and vehicle-treated animals; 10–12 sections per animal were analyzed. Cell counting and the area of the cortex were measured with ImageJ, and the values were expressed as cell density (microglia per mm^2^).

To quantify the compact Aβ plaques in the cortex and hippocampus of *Irf8*/5xFAD mice, 5–6 brain sections per mouse were acquired with the Zeiss fluorescence microscope, and the number of Thiazine Red-positive plaques in the region of interest was counted manually with ImageJ.

For quantification of Aβ inside microglia of *Irf8*/5xFAD mice, three brain sections per mouse were aquired with an Olympus confocal microscope at ×20 magnification, and the amount of internalized Aβ was quantified using ImageJ.

For quantification of Aβ internalized by isolated microglia, immunolabeled cells were imaged with the Zeiss fluorescence microscope; the number of Aβ spots inside each cell and the percentage of Aβ-containing microglia were manually counted in ImageJ.

### Immunoblot analysis of grafts

The cortical cell suspension was centrifuged at 800*g* for 5 min at 4 °C, and the pellet was lysed by resuspension in RIPA buffer (1 ml per 1 × 10^7^ cells). After 30-min incubation on ice, the samples were centrifuged at 13,000*g* for 10 min at 4 °C, and the supernatant was stored at −20 °C until use. Brain homogenate of a 12-month-old 5xFAD mouse was prepared as previously described^10^. The total protein concentration was determined using a Pierce BCA Protein Assay Kit (Thermo Fisher Scientific). For separation, samples were mixed with NuPAGE Sample Reducing Agent (10×) and NuPAGE LDS Sample Buffer (4×) and loaded onto NuPAGE 4–12% Bis-Tris Mini Gels (Invitrogen).

Proteins were transferred onto PVDF membranes (Bio-Rad) and incubated with specific antibodies against Aβ (mouse, 1:1,000, Covance, 6E10), DCX (rabbit, 1:2,000, Abcam, ab18723), GFAP (rabbit, 1:2,000, Dako, Z033401-2), IBA1 (rabbit, 1:100, Wako, 016-20001), α-tubulin (chicken, 1:1,000; Abcam, ab89984) and β-actin–HRP (mouse, 1:3,000, Abcam, ab20272) and incubated with the corresponding HRP-linked secondary antibodies. Clarity Western ECL Substrate (Bio-Rad) was used for protein visualization. The blot was acquired with Image Lab 4 software (Bio-Rad).

### Laser lesion and in vivo imaging of microglia process outgrowth

Animals were anesthetized via i.p. injection of ketamine (100 mg kg^−1^ body weight) and xylazine (10 mg kg^−1^ body weight), and a 3-mm cranial window was implanted over the somato‐sensory cortex of 12–14-week-old *Irf8*^+/+^, *Irf8*^+/−^ and *Irf8*^−/−^ mice. During the imaging session, the body temperature was monitored and maintained at 36–37 °C using a heating blanket. Depth of anesthesia was assessed by monitoring pinch withdrawal and vibrissae movements.

Laser lesion and acute two-photon imaging were carried out using Olympus FV1000 with Mai Tai DeepSee Laser (Spectra-Physics) with an excitation wavelength of 900 nm and an emission filter of 515–560 nm. Focused laser injury was induced ain a small area of about 80 μm^2^ within the region of interest at 50 μm below the pial surface using a laser pulse (75% power for 30 s) with a frequency of 8 μs per pixel. Microglia response was recorded every 5 min for a total of 35 min at a depth of 30–70 μm with 2.5-μm *z* increments and 512 × 512-pixel resolution. In each mouse, 4–6 different sites were recorded. At the end of the experiment, mice were killed by decapitation.

### Quantification of process microglia movement

The extension of individual microglia processes was tracked manually and measured using ImageJ considering the initial distance (at *t* = 0 min) and the final distance (at *t* = 35 min) from the center of the laser lesion. The mean speed and distance traveled were calculated for each microglial process. The percentage of responsive microglia cells was quantified by counting cells that showed at least one process moving toward the lesion site. The positions of the processes at *t* = 0 min were divided into four ranges: 0–30 µm, 31–60 µm, 61–90 µm and 91–120 µm (see the results above).

### Laser lesion and in vivo imaging of Aβ-containing microglia in *Irf8*^+/+^/*Cx3cr1*^+/−^/5xFAD and *Irf8*^−/−^/*Cx3cr1*^+/−^/5xFAD mice

*Irf8*^+/+^/*Cx3cr1*^+/−^/5xFAD and *Irf8*^−/−^/*Cx3cr1*^+/−^/5xFAD mice (7–10 months old) were anesthetized via i.p. injection of ketamine (100 mg kg^−1^ body weight) and xylazine (10 mg kg^−1^ body weight), and a craniotomy was performed as described above, but before covering the exposed cortex with the glass coverslip, the dura was carefully removed, and 10 μl of 0.01% Thiazine Red diluted in sterile 1× PBS was applied to the cerebral cortex for 5 min.

Laser lesion and acute two-photon imaging were carried out as described above with the following main differences: (1) laser injury was induced in a larger area of about 2 mm^2^ within the region of interest; (2) emission filters of 590–650 nm and 515–560 nm were used to detect, respectively, Thiazine Red and GFP signal; and (3) microglia response was recorded every hour for a total of 6 h on the first day at a depth of 0–70 μm. Imaging of the same lesioned region was carried out, as a single acquisition, 24 h later. At the end of the experiment, the mouse was killed by decapitation.

Aβ area and microglia number were quantified, using ImageJ, after lesion induction (*t =* 0 h), at the end of the acute imaging session (*t* = 6 h) and after 24 h (*t* = 24 h) in a defined area of 150 µm × 150 µm around the lesion, to calculate the Aβ and microglia movement over time between the two groups.

### In vivo Aβ phagocytosis assay

5xFAD mice (young adults: 10–12 weeks old; adults: 10–12 months old) were injected intraperitoneally with methoxy-XO4 (10 mg kg^−1^ body weight, Tocris, cat. no. 4920). After 3 h, mice were transcardially perfused with ice-cold 1× PBS. Hippocampi were collected, and microglia were isolated by using density gradient separation and prepared as described previously with slight modifications^10^. In addition to the microglia surface markers CD11b (1:200, clone M1/70, BioLegend, cat. no. 101212) and CD45 (1:200, clone 30-F11, BioLegend, cat. no. 103106), the following lineage markers were added: anti-CD3 (1:300, clone 17A2, BioLegend, cat. no. 100220), anti-CD19 (1:300, clone 6D5, BioLegend, cat. no. 115520), anti-CD45R (1:300, clone RA3-6B2, BD Biosciences, cat. no. 552772), Ly6C (1:300, clone AL-21, BD Biosciences, cat. no. 560593) and Ly6G (1:300, clone 1A8, BD Biosciences, cat. no. 560601) for 20 min at 4 °C. Percentage and MFI of methoxy-XO4-positive CD11b^+^CD45^low^ microglia were determined by flow cytometry using a FACSCanto II (BD Biosciences) and analyzed with FlowJo software (Tree Star).

### RNA isolation from isolated microglia

Grafts were isolated from the cortex of 5xFAD mice 4 weeks after injection. In brief, mice were overdosed through i.p. injection of ketamine and xylazine and transcardially perfused with 10 ml of ice-cold PBS. Brains were immediately isolated and divided coronally at the injection site. The cortical grafts were identified under a stereo-microscope and carefully isolated with a small scalpel; as control, small cortical pieces near the transplant were also isolated. The cortical samples were collected in FACS medium and maintained on ice until further use.

Microglia were isolated and gated as described above. CD11b^+^CD45^low^ microglia were directly sorted by FACS into cell lysis buffer with MoFlo Astrios (Beckman Coulter). Total RNA extraction using Arcturus PicoPure RNA Isolation Kit (Thermo Fisher Scientific, cat. no. KIT0204) was performed according to the manufacturer’s protocol.

### Bulk RNA-seq from isolated microglia

The SMART-Seq v4 Ultra Low Input RNA Kit for Sequencing (Clontech) was used to generate first-strand cDNA from 300 pg of totalRNA. Double-stranded cDNA was amplified by long-distance PCR (13 cycles) and purified via magnetic bead clean-up. Library preparation was carried out as described in the Illumina Nextera XT Sample Preparation Guide. Next, 150 pg of input cDNA was tagmented (tagged and fragmented) by the Nextera XT transposome. The products were purified and amplified via a limited-cycle PCR program to generate multiplexed sequencing libraries. For the PCR step, 1:5 dilutions of index 1 (i7) and index 2 (i5) primers were used. The libraries were quantified using the KAPA Library Quantification Kit—Illumina/ABI Prism User Guide (Roche Sequencing Solutions). Equimolar amounts of each library were sequenced on a NextSeq 500 instrument controlled by the NextSeq Control Software, version 2.2.0, using two High Output Kits (75 cycles) with the dual index, single-read run parameters. Image analysis and base calling were done by the Real Time Analysis Software, version 2.4.11. The resulting BCL files were converted into FASTQ files with the bcl2fastq software, version 2.18.

Library preparation and RNA-seq were performed at the Genomics Core Facility ‘KFB - Center of Excellence for Fluorescent Bioanalytics’ (University of Regensburg; http://www.kfb-regensburg.de).

### Differential gene expression analysis

FASTQ files were aligned to the mouse genome (GENCODE M21 genome version and transcriptome annotation) using the STAR aligner (version 2.7.2a)^30^. Aligned BAM files were processed using featureCounts (version 1.6.2) from the subread package^31^ using standard settings to obtain gene counts.

Differential gene expression analysis was performed using the limma/voomWithQualityWeights pipeline from the R package loom^32^. Genes were deemed differentially expressed at an adjusted *P* of less than 0.01. For the volcano plot, data were plotted using R packages ggplot2 (version 3.3.3) and ggrepel (version 0.9.1). An expression heat map was generated from the expression data using the R package pheatmap^33^.

### Microglia depletion

CSF-1R inhibitor BLZ945 (Novartis) was dissolved in 20% (2-hydroxypropyl)-β-cyclodextrin (Sigma-Aldrich). A dose of 200 mg kg^−1^ body weight was used. In 9–10-week-old *Cx3cr1*^+/−^/5xFAD mice, BLZ945 was administered by oral gavage for 30 consecutive days starting 3 d before cell transplantation. The mice were killed and analyzed 1 d after the last application.

### Organotypic brain slice cultures

Organotypic brain slices from 9–11-month-old *Cx3cr1*^+/−^/5xFAD and P6–7 WT (C57BL6J) mice were prepared as previously described^20^. In brief, postnatal WT mice were killed by decapitation and aged mice by CO_2_ inhalation, following animal handling laws. After brain isolation, the olfactory bulb, midbrain, brain stem and cerebellum were removed, and the remaining cortical hemispheres were separated and cut sagittally at 350 μm using a tissue chopper (Mcllwain, model TC752, Mickle Laboratory Engineering). Intact cortico‐hippocampal slices from postnatal brains and cortical slices from aged brains were selected in a pre‐cooled dissection medium (50% HEPES‐buffered MEM, 1% penicillin–streptomycin, 10 mM Tris, pH 7.2) and incubated in the same medium for 30 min at 4 °C. Two postnatal cortico-hippocampal slices were plated together with two aged cortical slices onto a 0.4‐μm porous polytetrafluoroethylene membrane insert (PICMORG50, Millipore) placed in a 3.5-cm dish containing slice culture medium (50% HEPES‐buffered MEM, 25% HBSS, 1 mM l‐glutamine (Gibco) and 25% heat‐inactivated horse serum (Merck‐Sigma)). A minimum of nine co-culture dishes were prepared using one aged *Cx3cr1*^+/−^/5xFAD mouse per experiment (*n* = 2). Medium was exchanged 1 d after preparation and subsequently every 3–4 d. Slices were kept in culture for 2 weeks and then fixed in 4% PFA/sucrose for 15 min at room temperature, followed by three washes with PBS. For the immunohistochemical analysis of co-cultures, slices were permeabilized in PBS containing 0.5% Triton X‐100 (PBS-T) for 30 min, cut from the insert using a scalpel and subsequently incubated with the blocking solution (5% NGS in PBS-T) for 1 h at room temperature, followed by overnight incubation at room temperature with the primary anti-GFP antibody (1:500, Fitzgerald, cat. no. 20R‐GR011) added to the blocking solution. Slices were washed three times for 10 min with PBS-T and incubated for 3 h at room temperature with blocking solution containing the secondary antibody goat anti-rabbit Alexa Fluor 488 (1:250) (Life Technologies) and Hoechst 33342 for nucleus visualization (1:1,000, cat. no. H3570, Thermo Fisher Scientific). Slices were washed three times for 10 min with PBS-T and subsequently stained with Thiazine Red (Sigma‐Aldrich, 2 μM solution in PBS) for 20 min in the dark at room temperature, followed by three PBS-T washing steps. Slices were mounted using Gel Aqua Mount medium (Sigma‐Aldrich) and analyzed by confocal microscopy (Leica TCS SP5 II). Representative overview images were acquired with a 10× objective and high-magnification images with a 63× water immersion objective.

### Statistical analysis

GraphPad Prism 7 was used for statistical analysis. Student’s *t*-test, Mann–Whitney test, Kruskal–Wallis test followed by Dunn’s multiple comparison test or one-way or two-way ANOVA followed by Sidak’s, Dunn’s or Tukey’s multiple comparison test was applied. For correlation, the Pearson correlation was used. Reported values are means ± s.e.m. Significance level *α* was set to 0.05; *P < 0.05, **P < 0.01, ***P < 0.001 and ****P < 0.0001. No statistical methods were used to predetermine sample sizes, but our sample sizes are similar to those reported in previous publications^5,13^. Data distribution was assumed to be normal, but this was not formally tested. Investigators were blinded for staining experiments, microglia counts, assessment of Aβ and flow cytometry experiments.

### Reporting Summary

Further information on research design is available in the Nature Research Reporting Summary linked to this article.

## Online content

Any methods, additional references, Nature Research reporting summaries, source data, extended data, supplementary information, acknowledgements, peer review information; details of author contributions and competing interests; and statements of data and code availability are available at 10.1038/s41593-021-00951-0.

## Supplementary information

Supplementary Information Supplementary Figs. 1–3, Supplementary Fig. legends and legends for Supplementary Videos 1–6

Reporting Summary

Supplementary Video 1 Video file showing microglia in vivo under resting state condition in an *Irf8*^+/−^ mouse. Scale bar, 10 µm.

Supplementary Video 2 Video file showing microglia in vivo under resting state condition in an *Irf8*^−/−^ mouse. Scale bar, 10 µm.

Supplementary Video 3 Video file showing microglial responses to a localized laser-induced injury in vivo in an *Irf8*^+/−^ mouse. Scale bar, 15 µm.

Supplementary Video 4 Video file showing microglial responses to a localized laser-induced injury in vivo in an *Irf8*^−/−^ mouse. Scale bar, 15 µm

Supplementary Video 5 Video file showing an Aβ-containing microglial cell moving toward the lesion. Scale bar, 20 µm.

Supplementary Video 6 Video file showing Aβ-containing microglia moving toward the lesion. Scale bar, 20 µm.

## Data Availability

The data that support the findings of this study are available from the corresponding author upon reasonable request. The raw data for RNA sequences of this project are available at the Gene Expression Omnibus under accession code GSE162920. Source data are provided with this paper.

## Source data

Source Data Fig. 1 Statistical Source Data
Source Data Fig. 2 Statistical Source Data
Source Data Fig. 3 Statistical Source Data
Source Data Extended Data Fig. 1 Statistical Source Data
Source Data Extended Data Fig. 2 Unprocessed Western Blots
Source Data Extended Data Fig. 3 Statistical Source Data
Source Data Extended Data Fig. 4 Statistical Source Data
Source Data Extended Data Fig. 5 Statistical Source Data
Source Data Extended Data Fig. 6 Statistical Source Data
Source Data Extended Data Fig. 7 Statistical Source Data
Source Data Extended Data Fig. 8 Statistical Source Data

## Code availability

The code used to analyze these data are available from the corresponding author upon reasonable request.
