# Supplementary material for: Microglia contribute to the propagation of Aβ into unaffected brain tissue
Source: Nat Neurosci. 2021 Nov 22;25(1):20–5. doi: 10.1038/s41593-021-00951-0 (PMC8737330; doi:10.1038/s41593-021-00951-0)
Supplement: Supplementary file 1 — Supplementary Figs. 1–3, Supplementary Fig. legends and legends for Supplementary Videos 1–6 [file 41593_2021_951_MOESM1_ESM.pdf]

nature portfolio

https://doi.org/10.1038/s41593-021-00951-0

Supplementary information

Microglia contribute to the propagation of Aβ into unaffected brain tissue

In the format provided by the authors and unedited

Microglia contribute to the propagation of Aβ into unaffected brain tissue

Paolo d'Errico, Stephanie Ziegler-Waldkirch, Vanessa Aires, Philippe Hoffmann, Charlotte Mezö, Daniel Erny, Laura Sebastian Monasor, Sabine Liebscher, Vidhya M Ravi, Kevin Joseph, Oliver Schnell, Katrin Kierdorf, Ori Staszewski, Sabina Tahirovic, Marco Prinz and Melanie Meyer-Luehmann*

***Corresponding author :** melanie.meyer-luehmann@uniklinik-freiburg.de

This PDF file includes:

Supplementary Data Fig. 1 to 3

Captions for Supplementary Videos S1 to S6

Additional supplementary material for this manuscript includes the following:

Supplementary Videos S1 to S6

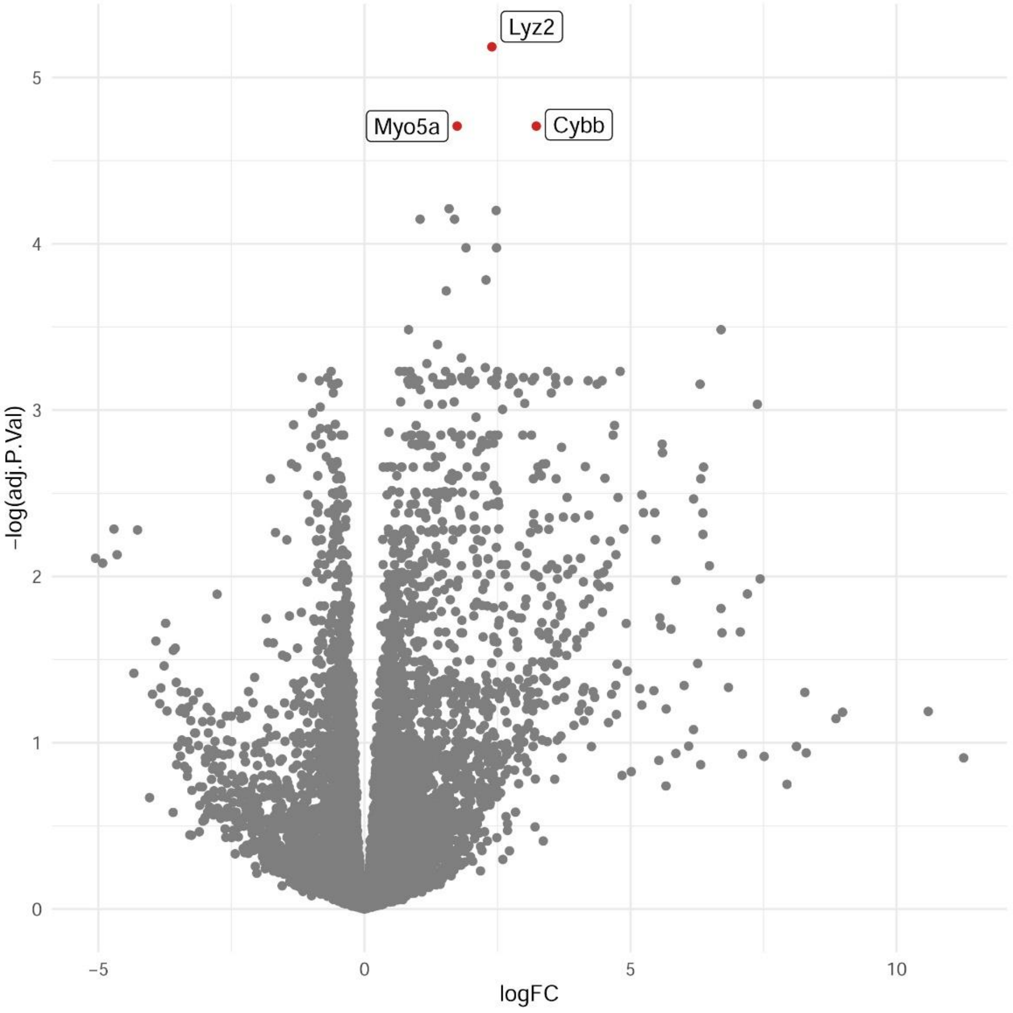

Supplementary Figure 1. Volcano plot of differential gene expression analysis results. Labeled and red colored genes are significantly dysregulated between graft and control tissue at a multiple testing adjusted p<0.01. Differentially expressed genes were determined using the Limma-voom package in R.

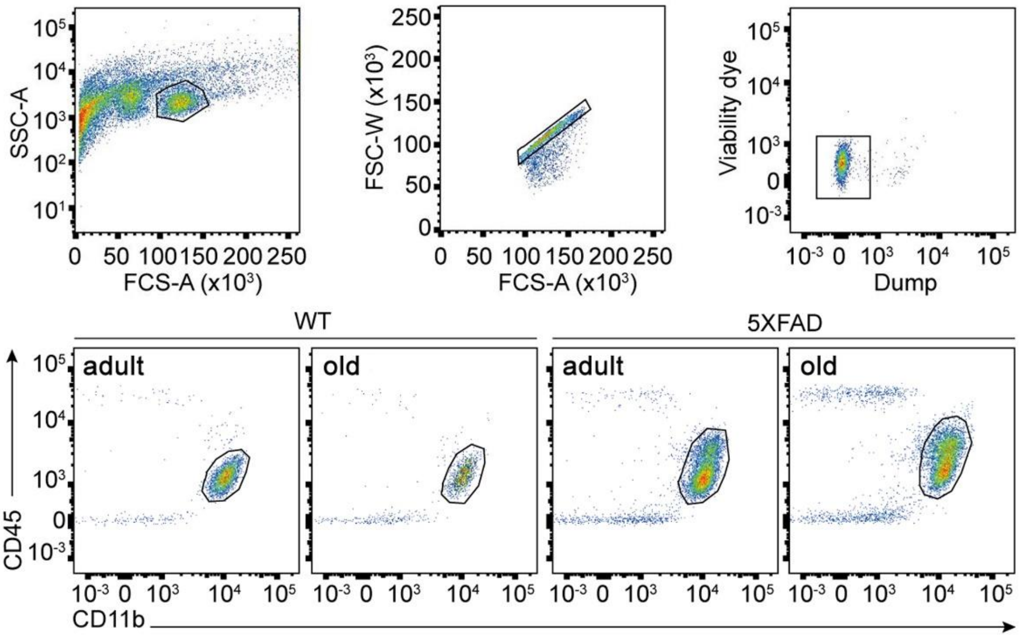

Supplementary Figure 2. FACS gating strategy. Gating strategy for flow cytometric analysis of single viable CD11b$^+$ CD45^{lo} methoxy-X04$^-$ and single viable CD11b$^+$ CD45^{lo} methoxy-X04$^+$ microglia from adult and old WT and 5xFAD mice.

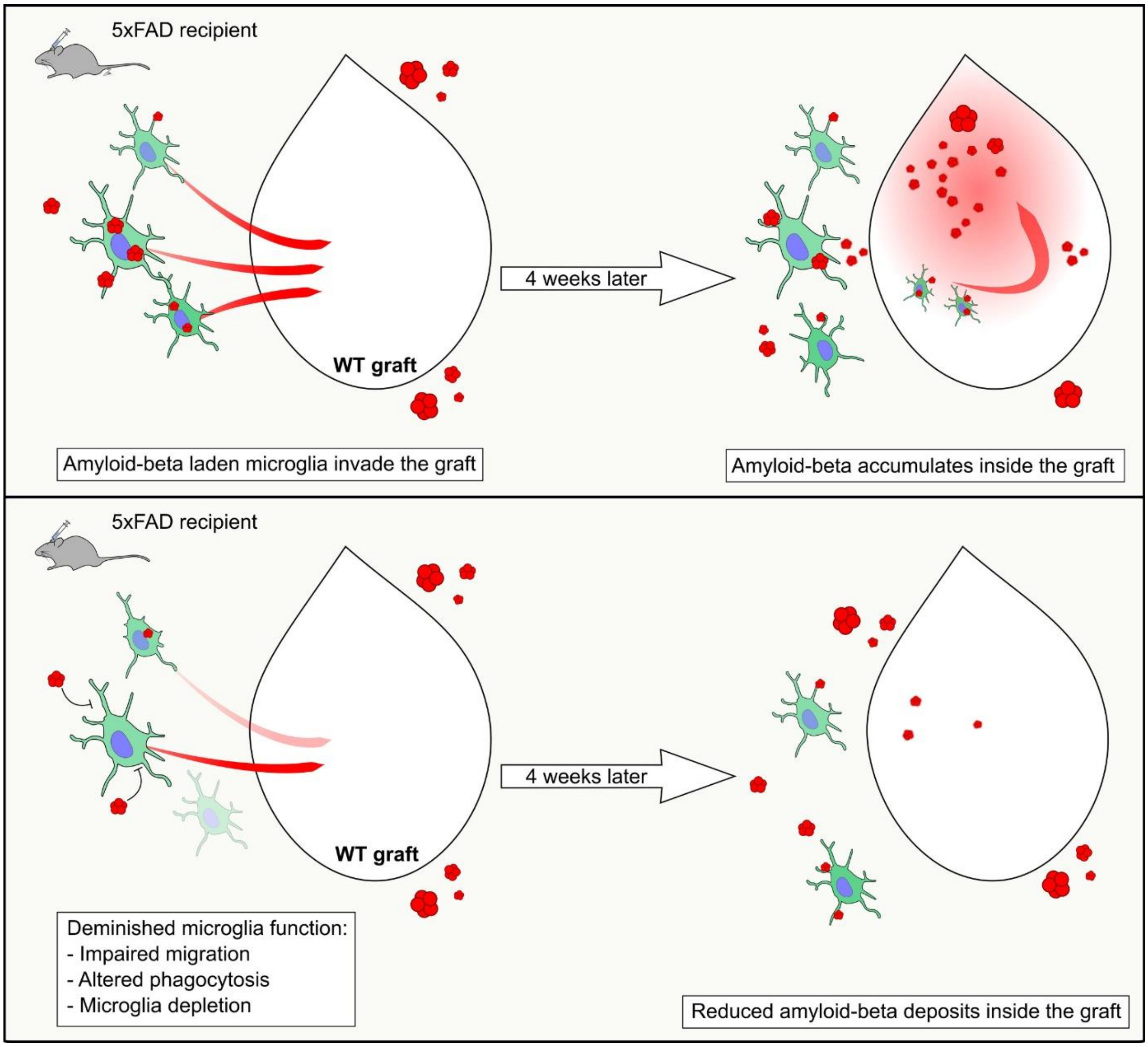

Supplementary Figure 3. Schematic overview of Aβ propagation involving microglia. Upper panel: Host microglia containing Aβ invade the WT graft in 5xFAD recipient mice leading to Aβ accumulation within the transplant. Lower panel: Manipulation of microglial function such as migration and phagocytosis or their elimination is accompanied by a reduction of Aβ load in the WT graft.

Supplementary Videos 1-6:

Video 1: Video file showing microglia *in vivo* under resting state condition in an $Irf8^{+/-}$ mouse. Scale bar, 10 µm.

Video 2: Video file showing microglia *in vivo* under resting state condition in an $Irf8^{-/-}$ mouse. Scale bar, 10 µm.

Video 3: Video file showing microglial responses to a localized laser-induced injury *in vivo* in an $Irf8^{+/-}$ mouse. Scale bar, 15 µm.

Video 4: Video file showing microglial responses to a localized laser-induced injury *in vivo* in an $Irf8^{-/-}$ mouse. Scale bar, 15 µm.

Video 5: Video file showing an Aβ-containing microglial cell moving towards the lesion. Scale bar, 20 µm.

Video 6: Video file showing Aβ-containing microglia moving towards the lesion. Scale bar, 20 µm.
