# Supplementary material for: Microglia contribute to the propagation of Aβ into unaffected brain tissue
Source: Nat Neurosci. 2021 Nov 22;25(1):20–5. doi: 10.1038/s41593-021-00951-0 (PMC8737330; doi:10.1038/s41593-021-00951-0)
Supplement: Supplementary file 2 — Reporting Summary [file 41593_2021_951_MOESM2_ESM.pdf]

nature research

Corresponding author(s): Melanie Meyer-Luehmann

Last updated by author(s): Aug 31, 2021

Reporting Summary

Nature Research wishes to improve the reproducibility of the work that we publish. This form provides structure for consistency and transparency in reporting. For further information on Nature Research policies, see Authors & Referees and the Editorial Policy Checklist.

Statistics

For all statistical analyses, confirm that the following items are present in the figure legend, table legend, main text, or Methods section.

n/a	Confirmed	
☐	☒	The exact sample size (*n*) for each experimental group/condition, given as a discrete number and unit of measurement
☐	☒	A statement on whether measurements were taken from distinct samples or whether the same sample was measured repeatedly
☐	☒	The statistical test(s) used AND whether they are one- or two-sided<br>*Only common tests should be described solely by name; describe more complex techniques in the Methods section.*
☐	☒	A description of all covariates tested
☐	☒	A description of any assumptions or corrections, such as tests of normality and adjustment for multiple comparisons
☐	☒	A full description of the statistical parameters including central tendency (e.g. means) or other basic estimates (e.g. regression coefficient) AND variation (e.g. standard deviation) or associated estimates of uncertainty (e.g. confidence intervals)
☐	☒	For null hypothesis testing, the test statistic (e.g. *F*, *t*, *r*) with confidence intervals, effect sizes, degrees of freedom and *P* value noted<br>*Give P values as exact values whenever suitable.*
☒	☐	For Bayesian analysis, information on the choice of priors and Markov chain Monte Carlo settings
☒	☐	For hierarchical and complex designs, identification of the appropriate level for tests and full reporting of outcomes
☐	☒	Estimates of effect sizes (e.g. Cohen's *d*, Pearson's *r*), indicating how they were calculated

Our web collection on statistics for biologists contains articles on many of the points above.

Software and code

Policy information about availability of computer code

Data collection	Fluorescently labeled brain slices or cell cultures were acquired using a Zeiss fluorescent microscope (Axio Imager M2M) or an Olympus confocal microscope (Fluoview FV 1000). Organotypic brain slices were acquired using a confocal microscopy (Leica TCS SP5 II).<br>In vivo imaging was performed with an Olympus FV1000 two-photon microscope with Mai Tai DeepSee Laser (Spectra-Physics, Newport Corporation, Franklin, MA, USA). 3D reconstruction was performed using IMARIS 8 software. Western blot membranes were visualized with ImageLab 4 software (Bio-Rad Laboratories).
Data analysis	ImageJ 1.52a was used for all immunohistochemical analyses and for quantification of two-photon in vivo acquisitions. The cDNAs libraries for Bulk-RNA sequencing were quantified using the KAPA Library Quantification Kit - Illumina/ABI Prism User Guide (Roche Sequencing Solutions, Inc., Pleasanton, CA, USA) and image analysis done by the Real Time Analysis Software (RTA) v2.4.11. Each library was sequenced on a NextSeq 500 instrument controlled by the NextSeq Control Software (NCS) v2.2.0. The resulting .bcl files were converted into .fastq files with the bcl2fastq v2.18 software.<br>For the analysis of flow cytometry experiments the FlowJo software (Tree Star) was utilized.<br>GraphPad Prism 7 (GraphPad Software, Inc) was used for statistical analysis and to generate graphs presented throughout the manuscript.

For manuscripts utilizing custom algorithms or software that are central to the research but not yet described in published literature, software must be made available to editors/reviewers. We strongly encourage code deposition in a community repository (e.g. GitHub). See the Nature Research guidelines for submitting code & software for further information.

Data

Policy information about availability of data

All manuscripts must include a data availability statement. This statement should provide the following information, where applicable:

- Accession codes, unique identifiers, or web links for publicly available datasets
- A list of figures that have associated raw data
- A description of any restrictions on data availability

All relevant data are available from the authors upon request.

Field-specific reporting

Please select the one below that is the best fit for your research. If you are not sure, read the appropriate sections before making your selection.

☒ Life sciences ☐ Behavioural & social sciences ☐ Ecological, evolutionary & environmental sciences

For a reference copy of the document with all sections, see nature.com/documents/nr-reporting-summary-flat.pdf

Life sciences study design

All studies must disclose on these points even when the disclosure is negative.

Sample size	Sample size was determined based on experience from previous studies (Meyer-Luehmann et al. Nature Neuroscience 2003; Meyer-Luehmann et al. Nature 2008), including the success rate of intracerebral grafting and two-photon imaging.
Data exclusions	Data were excluded if intracerebral grafting was unsuccessful and no graft was present; The number of mice per genotype were excluded as follows: 5xFAD/Cx3cr1+/-=5; 5xFAD/Cx3cr1-/-=3; old recipients 5xFAD/Cx3cr1+/-=4; 5xFAD/Irf8+/-=1; 5xFAD/CCR2+/-=3; 5xFAD/CCR2-/-=3.
Replication	All attempts of replication were successful.To analyze Aβ inside primary microglia, between 20 and 50 cells per mouse were quantified and replicated on at least 3 mice per group. The western blot analysis of freshly isolated cortical cells for grafting were performed on three independent preparations. Methoxy-XO4 FACS analysis of microglia isolated from young-adult and aged 5xFAD mice was performed in three repeated experiments using at least 3 mice per group in each experiment. For the Bulk-RNA sequencing, graft and control cortical region isolation was repeated three times and for each independent experiment 7-10 mice were used. The grafting experiments were repeated at least three times using, when possible, at least 2 mice per group per experiments. The organotypic brain slice experiments where replicated two times using one mouse per group in each experiment. The in vivo laser lesion experiments were performed in at least 4 repeated experiments per animal group using one mouse in each experiment.
Randomization	For all experiments, mice were randomly allocated into each experimental group by PdE and CM.
Blinding	The investigator was not blinded during the intracerebral grafting experiments since the procedure was independently identical. The investigator was not blinded during two-photon imaging and analysis due to obvious differences in microglia morphology between groups/ genotypes. The investigator was blinded for staining experiments, microglia counts and assessment of Aβ (slides and in vitro samples were imaged and saved with random numbers to identify them) and flow cytometry experiments. Images were then quantified and unblinded to perform group statistics.

Reporting for specific materials, systems and methods

We require information from authors about some types of materials, experimental systems and methods used in many studies. Here, indicate whether each material, system or method listed is relevant to your study. If you are not sure if a list item applies to your research, read the appropriate section before selecting a response.

Materials & experimental systems

n/a	Involved in the study
☐	☒ Antibodies
☒	☐ Eukaryotic cell lines
☒	☐ Palaeontology
☐	☒ Animals and other organisms
☒	☐ Human research participants
☒	☐ Clinical data

Methods

n/a	Involved in the study
☒	☐ ChIP-seq
☐	☒ Flow cytometry
☒	☐ MRI-based neuroimaging

Antibodies

Antibodies used	Iba1 Abcam, EPR16588 #ab178846, lot. GR3235737-5; Iba1 Wako #019-19741, lot.PTN5930 or #016-20001, lot.PDF3117; GFAP,

	clone GA5, Sigma-Aldrich #G3893, lot.3140011; anti-GFAP Dako #Z033401-2, lot.00087879; anti-Aβ clone 6E10 BioLegend #803001-Previously Covance catalog# SIG-39320, lot.B291304; DCX Abcam #ab18723, lot.GR324493-1; α-tubulin Abcam #ab89984, lot.GR3281114-3; β-actin-HRP Abcam #ab20272, lot.GR3256256-1; CD11b-APC, clone M1/70, Biolegend #101212, lot.1912131; anti-GFP, Fitzgerald, cat.no. 20R-GR011; CD45-PE, clone 30-F11, Biolegend #103106, lot.4348413; anti-CD3-PE/Cy7, clone 17A2, Biolegend #100220, lot.B266245; CD19-PE/Cy7, clone 6D5, Biolegend, #115520, lot.B290857; CD45R/B220-PE/Cy7, clone RA3-6B2, BD Bioscience #552772, lot.2008222; Ly6C-PE/Cy7, clone AL-21, BD Bioscience #560593, lot.9080848; Ly6G-PE/Cy7, clone 1A8, BD Bioscience, #560601, lot.8164873, Fixable Viability Dye eFluor® 780, eBioscience #65086514, lot.2114524.
Validation	All antibodies used in this study were commercially available and validated by the vendors and previous studies performed by our laboratory or by others.

Animals and other organisms

Policy information about studies involving animals; ARRIVE guidelines recommended for reporting animal research

Laboratory animals	Grafting experiments:<br>9-10 weeks or 8-11 months female mus musculus 5xFAD (APP K670N/M671L (Sw)+I716V (Fl)+V717I(Lo) and PS1M146L+L286V) crossed with Cx3cr1+/- in order to obtain Cx3cr1+/-/5xFAD or Cx3cr1-/-/5xFAD; 9-10 weeks female Hexb-tdTomato/5xFAD; Thy1-eGFP/5xFAD; Irf8+/+/Cx3cr1+/- /5xFAD, Irf8+/-/Cx3cr1+/-/5xFAD or Irf8-/-/Cx3cr1+/-/5xFAD; CCR2+/-/5xFAD or CCR2-/-/5xFAD. All mice were maintained on a C57BL/6N background.<br>Cortical suspension were prepared from WT C57BL/6N embryos (E16-E17). All embryos were used.<br><br>Microglia isolation experiments:<br>Microglia were isolated from 20-30 weeks or 50-60 weeks 5xFAD mice and aged matched C57BL/6N WT controls. Both males and females were used for these experiments.<br><br>Laser lesion and in vivo imaging of microglia process outgrowth:<br>12-14 weeks old female Irf8+/+/Cx3cr1+/-, Irf8+/-/Cx3cr1+/- and Irf8-/-/Cx3cr1+/- mice.<br><br>Laser lesion and in vivo imaging of Aβ–containing microglia in Cx3cr1+/-/5xFAD and Irf8-/-/Cx3cr1+/-/5xFAD mice:<br>7-10 months old female Cx3cr1+/-/5xFAD and Irf8-/-/Cx3cr1+/-/5xFAD mice.<br><br>Organotipic slice co-cultures:<br>9 months old Cx3cr1+/-/5xFAD and 6days old C57BL/6N WT mice.
Wild animals	No wild animals were used in the study.
Field-collected samples	No field-collected samples were used in the study.
Ethics oversight	All animal experiments were carried out in accordance with the policies of the state of Baden-Württemberg under license number G16-100.

Note that full information on the approval of the study protocol must also be provided in the manuscript.

Flow Cytometry

Plots

Confirm that:

☒ The axis labels state the marker and fluorochrome used (e.g. CD4-FITC).

☒ The axis scales are clearly visible. Include numbers along axes only for bottom left plot of group (a 'group' is an analysis of identical markers).

☒ All plots are contour plots with outliers or pseudocolor plots.

☒ A numerical value for number of cells or percentage (with statistics) is provided.

Methodology

Sample preparation	Mice were injected intraperitoneally with methoxy-XO4 (Tocris Bioscience; #4920; 10mg/kg bodyweight). Upon 3 hours incubation, mice were transcardially perfused with PBS. Hippocampi were collected and homogenized with a glass potter in HBSS containing 15 mM HEPES buffer and 0.54 % Glucose. Hippocampal brain homogenate was separated by 70/37/30 % layered Percoll gradient centrifugation at 800 g for 30 min at 4 °C (without brake). Subsequently, cell pellet was collected and washed once with PBS before staining. Cells were incubated with Fc receptor blocking antibody CD16/CD32 (2.4G2, BD Bioscience) to prevent unspecific binding and dead cells were stained using the Fixable Viability Dye eFluor® 780 (eBioscience) for 10min at 4°C. Cells were stained with primary antibodies directed against CD11b-APC (M1/70, BioLegend), CD45-PE (30-F11, BioLegend, Lineage markers: anti-CD3-PE/Cy7 (17A2, BioLegend), anti-CD19-PE/Cy7 (6D5, BioLegend), anti-CD45R-PE/Cy7 (RA3-6B2, BD Bioscience), Ly6C-PE/Cy7 (AL-21, BD Bioscience), Ly6G-PE/Cy7 (1A8, BD Bioscience) for 20min at 4°C.
Instrument	FACS Canto II (BD Bioscience)

Software	Data were acquired with BD FACSDiva™ SOFTWARE (Becton Dickinson). Post-acquisition analysis was performed using FlowJo software, version X.0.7.
Cell population abundance	Gating on live CD11b+CD45loDUMP- cells results in >95% pure microglial populations, while blood-derived cells and CAMs are excluded.
Gating strategy	The gating strategy for mouse brain samples we used in this study is provided in Supplementary Fig.4. CNS cells were gated for singlets, followed by gating for living cells (Fixable Viability Dye), lineage-negative cells (Dump), CD11b+CD45+ cells and methoxy-O4+ CD11b+CD45+ cells.

☒ Tick this box to confirm that a figure exemplifying the gating strategy is provided in the Supplementary Information.
