# Supplementary figures and images for: Microglia contribute to the propagation of Aβ into unaffected brain tissue
Source: Nat Neurosci. 2021 Nov 22;25(1):20–5. doi: 10.1038/s41593-021-00951-0 (PMC8737330; doi:10.1038/s41593-021-00951-0)

# Source Data

## Extended Data Figure 2

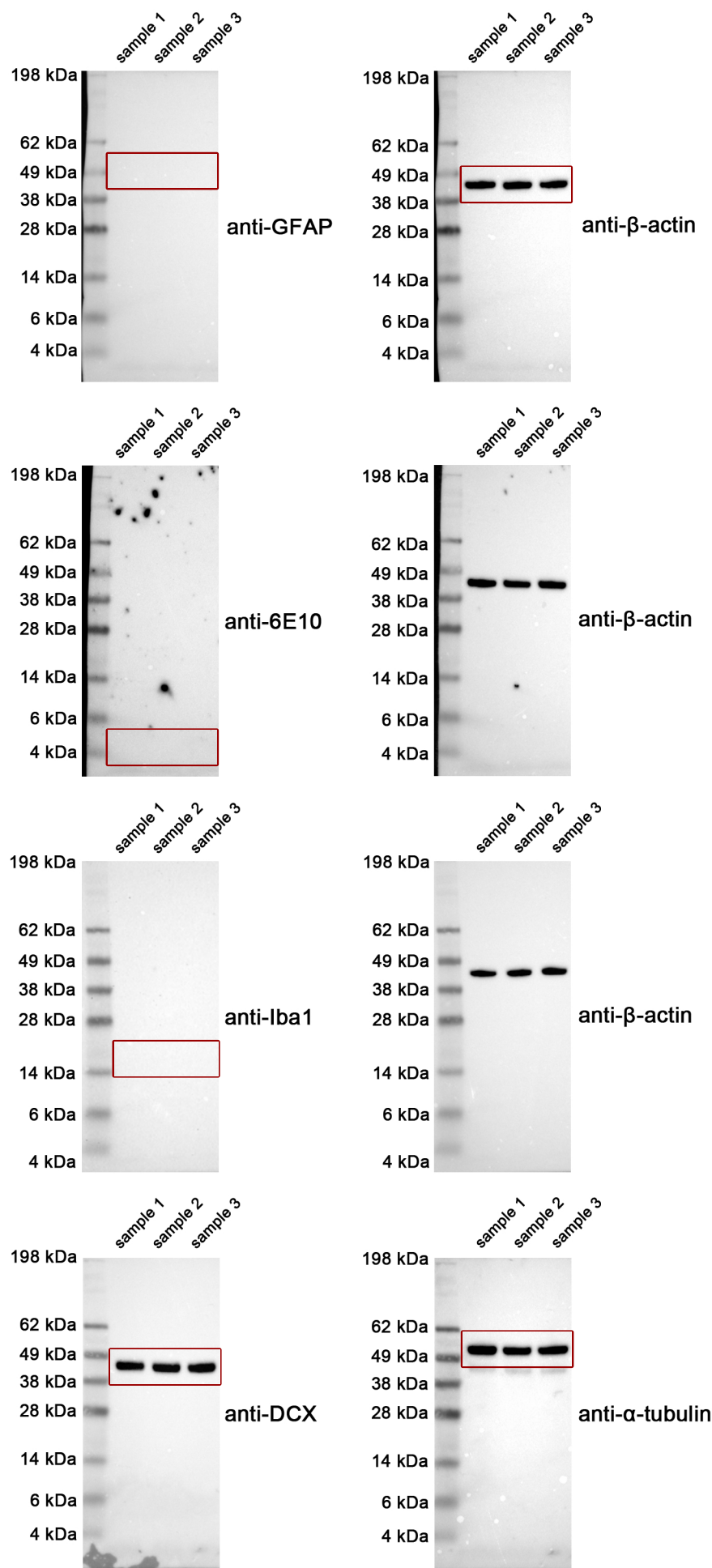

Supplement: Source Data Extended Data Fig. 2 — Unprocessed Western Blots [file 41593_2021_951_MOESM13_ESM.pdf]
